# Macrophage polarization: molecular mechanisms, disease implications, and targeted therapeutic strategies

**DOI:** 10.3389/fimmu.2025.1732718

**Published:** 2025-12-12

**Authors:** Yanan Ji, Xia Li, Xinlei Yao, Jiacheng Sun, Jia Yi, Yuntian Shen, Bingqian Chen, Hualin Sun

**Affiliations:** 1 Jiangsu Key Laboratory of Tissue Engineering and Neuroregeneration, Key Laboratory of Neuroregeneration of Ministry of Education, Co-innovation Center of Neuroregeneration, Nantong University, Nantong, Jiangsu, China; 2 Department of Orthopedics, Changshu Hospital Affiliated to Soochow University, First People’s Hospital of Changshu City, Changshu, Jiangsu, China

**Keywords:** macrophage polarization, molecular mechanisms, disease implications, therapeutic strategies, innate immunity

## Abstract

Macrophage polarization represents a fundamental plasticity process within innate immunity, profoundly influencing tissue homeostasis and disease progression. Based on developmental origins, macrophages are categorized into tissue-resident macrophages and monocyte-derived macrophages, which collectively form a dynamic host defense network. Notably, the functional states of macrophages exist along a continuum, extending beyond the classical pro-inflammatory (M1) and anti-inflammatory/reparative (M2) dichotomy. These states are dynamically shaped by spatiotemporally heterogeneous microenvironmental signals and coordinated through intricate molecular networks. Key signaling pathways guide polarization directions. Metabolic reprogramming, where M1 polarization relies on glycolysis and the pentose phosphate pathway while M2 polarization favors oxidative phosphorylation and fatty acid oxidation, not only supplies energy but also generates regulatory metabolites. Furthermore, epigenetic mechanisms, including DNA methylation, histone modifications, and non-coding RNAs, contribute to stabilizing polarized phenotypes. These mechanisms are interconnected, forming feedback loops that collectively sculpt macrophage functional diversity. Dysregulated polarization underlies numerous diseases. In response, therapeutic strategies targeting macrophage polarization are rapidly emerging. These include pharmacological interventions using small molecules and metabolic modulators to reprogram cell phenotypes, immunotherapies such as CAR-M macrophages or exosome-mediated reprogramming to remodel immune microenvironments, and precision regulation through gene editing or epigenetic modifications. Although innovations like single-cell omics, spatial transcriptomics, computational modeling, and synthetic biology are advancing the field, clinical translation still faces challenges including off-target effects, inefficient delivery, microenvironmental dependency. Future research must integrate multi-omics data to develop individualized therapies, further investigate the stability and plasticity of polarization states, and leverage smart materials and advanced model systems to advance precision immunotherapeutics.

## Graphical Abstract

**Figure:**
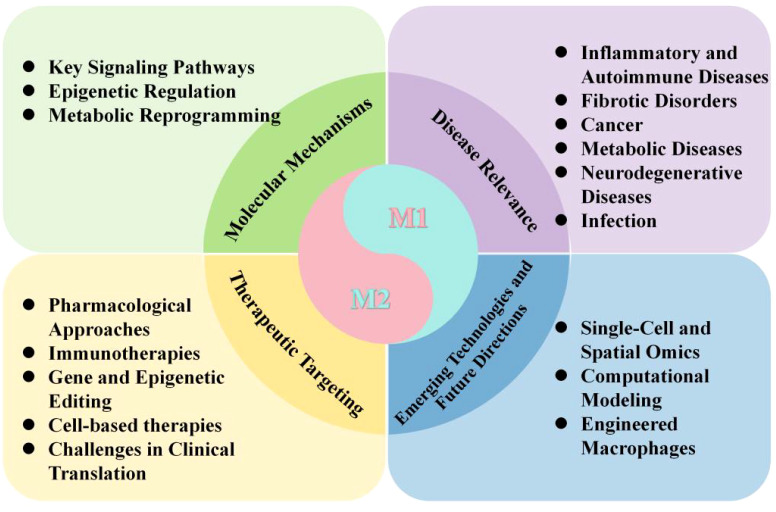

## 1 Introduction

### 1.1 Overview of macrophage biology

Macrophages are central components of the innate immune system and play key roles in maintaining tissue homeostasis regulating inflammatory responses and promoting tissue repair with their high heterogeneity and functional plasticity being the most distinctive features (1, 2). These cells are widely distributed across various tissues and organs and can respond to diverse microenvironmental signals exhibiting highly diverse phenotypes and functional states (3). Based on developmental origin and tissue localization macrophages are broadly categorized into two types tissue-resident macrophages (TRMs) and monocyte-derived macrophages (MDMs) (4). TRMs such as Kupffer cells in the liver and microglia in the central nervous system colonize tissues during early embryonic development primarily originating from the yolk sac or fetal liver and migrate to their final residence sites before birth. They maintain long-term residency mainly through local proliferation and perform organ-specific homeostatic functions including tissue repair and immune surveillance (3, 5–7). In contrast MDMs are continuously derived postnatally from hematopoietic stem cells in the bone marrow enter the circulation as monocytes and are recruited to sites of injury infection or inflammation (1, 3, 8). Macrophages residing in the organization, especially those in the red pulp and erythroid islands, are the core regulators of systemic and local iron homeostasis; They not only provide iron for red blood cell production by recycling aging red blood cells, but also directly release iron to neighboring hematopoietic cells (including erythroid precursor cells) through the iron export protein ferroportin, which is crucial for emergency hematopoiesis and maintaining the hematopoietic microenvironment (9, 10). Thus, both types of macrophages work in concert forming a critical and highly dynamic cellular network essential for host defense and tissue repair.

Macrophages exhibit remarkable functional diversity during development, homeostasis maintenance, tissue repair, and innate immunity due to their plasticity. This plasticity is traditionally described as a spectrum between two polarization states, namely classically activated pro-inflammatory M1 and alternatively activated anti-inflammatory or reparative M2 macrophages, which represent two extremes of functional phenotypes (1, 11, 12). However, single-cell omics studies have revealed that macrophage activation states form a complex and continuous functional spectrum rather than a simple binary model (13, 14). During tissue injury, macrophages encounter various signals in the microenvironment, including cytokines, chemokines, damage-associated molecular patterns (DAMPs), pathogen-associated molecular patterns (PAMPs), and particularly necrotic cell debris. These signals dynamically shape their polarization state over time and space (15–18). Subsequently, macrophages coordinate inflammatory progression and promote wound repair through the production of reactive oxygen species, proteases, cytokines, and chemokines. This polarization process is tightly regulated by multi-layered mechanisms. Key signaling pathways involve Toll-like receptors (TLRs), JAK/STAT, TGF-β/Smad, PPARγ, Notch, and multiple microRNA cascades (1, 13). Furthermore, epigenetic modifications such as histone lactylation and cellular metabolic reprogramming, including shifts between glycolysis and oxidative phosphorylation (OXPHOS), are recognized as critical intrinsic mechanisms determining macrophage functional states (13, 19, 20). Thus, through high plasticity and multi-level regulatory networks, macrophages perform diverse functions in complex physiological and pathological contexts while maintaining organismal homeostasis.

Under physiological conditions TRMs act as essential guardians of organ homeostasis by clearing apoptotic cells and debris regulating tissue regeneration maintaining metabolic balance and promoting immune tolerance (3, 21). For instance, intestinal macrophages are critical for epithelial barrier integrity and the stability of commensal microbiota (11, 21). However, under imbalanced microenvironments the recruitment activation and polarization of macrophages exhibit remarkable spatiotemporal heterogeneity and tissue-specificity profoundly influencing diverse disease processes (1, 11, 22, 23). In liver diseases including acute injury viral hepatitis alcohol- or metabolic-associated fatty liver disease fibrosis and hepatocellular carcinoma macrophage polarization particularly the crosstalk between Kupffer cells and MDMs plays a central role in disease initiation progression and resolution. These cells secrete factors such as TGF-β galectin-3 and interleukins (ILs) to regulate hepatic stellate cell activation and extracellular matrix deposition (1, 24). Similarly in fibrotic diseases like hepatic and renal fibrosis and within the tumor microenvironment (TME) macrophages and cancer-associated fibroblasts form complex communication networks through axes such as CSF1/CSF1R collectively driving fibrogenesis or supporting tumor growth angiogenesis immune evasion and metastasis (16, 22, 25). In chronic inflammatory conditions such as inflammatory bowel disease and non-healing wounds macrophage dysfunction—such as sustained pro-inflammatory activation or impaired repair function—is key to persistent tissue damage (11, 26). Even in bone remodeling and neural homeostasis specific macrophage subsets like osteomacs and microglia participate in fine-tuned regulation (6, 27). Notably TAMs within the TME display high heterogeneity and spatial distribution specificity. Different subsets such as pro-tumoral SPP1^+^ TAMs or potentially anti-tumoral IL4I1^+^/FOLR2^+^ TAMs localize to distinct niche regions perform divergent functions and are closely linked to prognosis (28, 29). This heterogeneity stems partly from developmental origins—tissue-resident versus monocyte-derived—and spatiotemporal variations in local microenvironments (14, 15). Thus, macrophages play multifaceted and central roles in both physiological and pathological processes. Their high plasticity and heterogeneity not only underpin tissue homeostasis but also deeply influence disease pathogenesis progression and outcome making them compelling therapeutic targets.

In summary, macrophages are central effectors of the innate immune system, and their functions are co-regulated by diverse developmental origins—such as TRMs and MDMs—along with high plasticity. Through the dynamic spectrum of M1/M2 polarization, macrophages precisely maintain tissue homeostasis and inflammatory balance. Under physiological conditions, they support barrier integrity and metabolic tolerance, while in pathological contexts, they deeply contribute to disease processes such as liver disorders, cancer, and fibrosis. This spatiotemporally specific multi-layered regulatory network underscores the potential of macrophages as crucial therapeutic targets.

### 1.2 Concept of macrophage polarization

Macrophage polarization is a plasticity process in which these cells adopt distinct functional states upon microenvironmental signals and it plays a central role in maintaining tissue homeostasis and driving disease progression (30). Traditionally, macrophage polarization is divided into two main phenotypes the classically activated pro-inflammatory M1 type and the alternatively activated anti-inflammatory or reparative M2 type. M1 macrophages are typically induced by signals such as interferon-gamma (IFN-γ) or lipopolysaccharide (LPS) and they highly express reactive oxygen species (ROS) and nitric oxide while secreting pro-inflammatory cytokines like TNF-α and IL-12 thereby contributing to pathogen clearance and anti-tumor immunity (31, 32). Conversely, M2 macrophages are mainly activated by factors such as IL-4 or IL-13 and they participate in tissue repair, angiogenesis, and immunoregulation through the secretion of mediators including arginase-1 (Arg1) and IL-10 thus exerting anti-inflammatory effects (33, 34). Therefore, the dynamic switching between M1 and M2 phenotypes allows macrophages to precisely regulate the balance between pro-inflammatory defense and anti-inflammatory repair which underlies their broad involvement in physiological homeostasis and various disease processes.

However, the traditional M1/M2 dichotomy oversimplifies the functional continuum of macrophages in complex biological environments. Advances in transcriptomic studies have provided growing evidence that macrophage activation states exist along a spectrum rather than falling into discrete categories. For instance, a large-scale transcriptomic analysis of 299 human macrophage samples under various stimulations revealed a continuous transitional activation landscape rather than clearly separated M1 or M2 poles, significantly expanding the conceptual boundaries of the classical binary model (35). The molecular basis of this continuous phenotypic switching involves the integration of complex signaling pathways such as TLRs, STAT proteins, and nuclear receptors, all of which are finely regulated by epigenetic modifications (13). Metabolic reprogramming is considered a central driver of macrophage plasticity, where specific microenvironmental signals induce distinct metabolic programs. M1 polarization is typically associated with enhanced aerobic glycolysis, upregulation of the pentose phosphate pathway, increased fatty acid synthesis, and rewiring of the tricarboxylic acid (TCA) cycle, often accompanied by suppressed mitochondrial respiration. In contrast, M2 polarization tends to enhance OXPHOS and FAO. These metabolic shifts directly influence the effector functions of macrophages (36, 37). Notably, pro-inflammatory activation does not entirely rely on OXPHOS suppression, and different stimuli can induce highly plastic metabolic phenotypes (38). Within the atherosclerotic plaque microenvironment, two distinct macrophage subsets, Mox and Mhem, have been identified, each driven by specific pathological stimuli and characterized by unique transcriptional and functional profiles (39). Mox macrophages are polarized by oxidized phospholipids, such as those found in oxidized low-density lipoprotein (oxLDL), primarily through the activation of the NRF2 signaling pathway (40). This phenotype is characterized by a high expression of antioxidant genes, including heme oxygenase-1 (HO-1) (41), and exhibits a distinctive iron-handling profile featuring increased iron storage alongside potentially compromised iron export, leading to intracellular iron retention (42). Functionally, Mox macrophages display impaired phagocytic capacity and undergo a metabolic shift from oxidative phosphorylation to glycolysis (43). In contrast, Mhem macrophages are induced by hemoglobin-haptoglobin complexes or heme released during intraplaque hemorrhage (40, 44). Their polarization is dependent on the AMPK-Activating Transcription Factor 1 (ATF1) signaling cascade (45, 46). Although Mhem cells also highly express HO-1 (41), they are functionally defined by their efficient promotion of cholesterol efflux, protection against foam cell formation, and anti-inflammatory properties, collectively conferring atheroprotective functions within the lesion (45, 46). Both the Mox and Mhem phenotypes diverge from the classical M1/M2 dichotomy, thereby contributing to the complex spectrum of macrophage heterogeneity in atherosclerosis (39, 44).Single-cell RNA sequencing (scRNA-seq) has revealed the existence of multiple functionally specialized subsets within tumor-associated macrophages (TAMs), including FCN1^+^, SPP1^+^, C1Q^+^, and CCL18^+^ TAMs, among others (47). Among these, FCN1^+^ TAMs, typically monocyte-derived and characterized by high expression of FCN1 and S100A family genes, exhibit a pro-inflammatory phenotype and are considered an early or intermediate state in TAM differentiation (47, 48). In contrast, SPP1^+^ TAMs represent a crucial subset defined by high expression of SPP1 (osteopontin) and are concurrently enriched for genes associated with lipid metabolism, immune regulation, and angiogenesis, such as APOE, TREM2, and VEGFA (47, 48). Functionally, SPP1^+^ TAMs play a central role in promoting tumor cell epithelial-mesenchymal transition (EMT), angiogenesis, intravasation, metastasis, and immunosuppression through the secretion of factors including SPP1, CCL18, CXCL8, TNF-α, and IL-1β. Their abundance is significantly correlated with poor prognosis in patients with various cancers, including head and neck squamous cell carcinoma (HNSCC), colorectal cancer (CRC), and liver cancer (48–50). Furthermore, C1Q^+^ TAMs demonstrate high expression of complement genes (e.g., C1QA, C1QB, C1QC) and antigen presentation-related genes, suggesting a role in immune regulation and suppression (47). CCL18^+^ TAMs are identified as terminally differentiated macrophages with potent immunosuppressive capabilities; the CCL18 they secrete can directly inhibit T cell function and enhance tumor metastatic potential (47). These findings delineate the remarkable heterogeneity of TAMs and define the distinct functions of individual subsets in shaping an immunosuppressive tumor microenvironment, driving cancer progression, and influencing responses to immunotherapy. Collectively, the functional states of macrophages extend beyond the simplistic M1/M2 framework, forming a multidimensional continuum shaped by signaling networks, epigenetic regulation, and metabolic reprogramming, which profoundly influences their diversity and adaptability in both physiological and pathological contexts.

The clinical significance of macrophage polarization states has been extensively validated across various diseases. In colorectal cancer, a high density of M2-like macrophages within the tumor stroma strongly correlates with reduced cancer-specific survival, while an elevated M1/M2 ratio indicates better prognosis, suggesting polarization status rather than overall density serves as a critical determinant of disease outcome (51). In osteoarthritis (OA), the polarization spectrum of synovial macrophages plays a central role in joint inflammation and disease severity; their phenotypes extend beyond the conventional M1/M2 classification, making targeted modulation of these cells a promising therapeutic strategy for OA (34). Furthermore, following central nervous system injury, the complex phenotypic continuum formed by microglia/macrophages dually regulates neuroregeneration, and functional imbalance within this system is considered a major cause of failed neural repair (52). Together, these findings underscore the prognostic value and therapeutic potential of macrophage polarization in oncology, joint disorders, and neural injury, and precise characterization of their continuous phenotypic spectrum will be essential for advancing diagnosis and treatment in these fields.

Macrophage polarization represents a core manifestation of functional plasticity, and its conceptual framework has evolved from the traditional M1/M2 dichotomy toward a spectrum theory. This process is precisely regulated by multiple signaling pathways, metabolic reprogramming, and epigenetic modifications. It dynamically balances pro-inflammatory defense and anti-inflammatory repair mechanisms, profoundly influencing the progression of various diseases. The clinical value and therapeutic potential demonstrated in conditions such as cancer, osteoarthritis, and neural injuries are now driving groundbreaking advances in multimodal precision-based diagnostic and therapeutic strategies targeting polarization spectra.

### 1.3 Physiological and pathological roles of macrophage

Macrophage polarization plays a central role in maintaining physiological homeostasis and regulating pathological processes, with its phenotypic plasticity directly influencing tissue homeostasis, immune defense, and repair mechanisms. Under physiological conditions, M2 macrophages contribute to tissue homeostasis by secreting anti-inflammatory and pro-repair factors such as IL-10 and TGF-β. For instance, during cardiac repair, RNF149 promotes post-infarction tissue remodeling through ubiquitin-mediated degradation of IFNGR1 to suppress excessive inflammation (53), while in muscle regeneration, Gasdermin D (GSDMD)-mediated metabolic reprogramming enables macrophages to release 11,12-EET, activating muscle stem cells and restoring function (54). In a variety of tumors (such as lung cancer and breast cancer), not only cancer cells themselves will produce EETs, but tumor related macrophages may also release EETs through mechanisms similar to GSDMD or other ways to promote cancer progression (55–57). Tissue repair typically follows a defined temporal sequence after skin or muscle injury, with an inflammatory phase lasting 1–2 days, a proliferative phase peaking within days, and a remodeling phase extending over months (58). In immune defense, macrophages undergo polarization shifts to combat pathogens. During antiviral responses, the aspartate-argininosuccinate shunt pathway generates fumarate, which enhances type I interferon production via MAVS protein succination (59). However, in systemic lupus erythematosus (SLE) patients, abnormal activation of the argininosuccinate synthase pathway in immune cells can trigger autoimmune responses and exacerbate damage to organs such as the skin and kidneys (60). In contrast, during sepsis, the ADAP/BTK/STAT3 axis induces a podoplaninhi macrophage subset that significantly augments phagocytic capacity to eliminate pathogens (61). The iron processing ability of macrophages is closely related to their polarization state: the pro-inflammatory M1 phenotype tends to retain iron by downregulating ferroportin to perform nutritional immunity and produce reactive oxygen species; The reparative M2 phenotype promotes iron release through ferroportin to support tissue repair and red blood cell generation (9, 62). Thus, through precise polarization control, macrophages act as pivotal regulators in tissue repair, temporal coordination of regeneration, and immune defense.

Wound healing relies on the timely transition of macrophages from an early pro-inflammatory M1 phenotype to a late reparative M2 phenotype. However, this transition is often dysregulated in chronic diseases such as diabetes, where persistent M1 polarization sustains a pro-inflammatory microenvironment and severely impairs healing. Novel biomaterials can restore polarization balance through multiple mechanisms. For example, a Filgotinib-loaded PEG hydrogel corrects glycolytic metabolism by inhibiting the JAK/STAT pathway, promotes OXPHOS, improves mitochondrial function, and accelerates skin healing (63). A silk fibroin hydrogel co-delivering FGF21 and H_2_S enables staged regulation, with early H_2_S release reducing inflammation and bacterial infection, followed by sustained FGF21 release to promote angiogenesis and M2 polarization (64). Plant-derived nanomaterials such as lemon exosome-based hydrogels, along with MMP-9-responsive smart hydrogels delivering M2 exosomes, can reprogram macrophage phenotypes and improve diabetic wound healing (65, 66). Furthermore, exosomes from healthy tendon stem cells can break the positive feedback loop between senescent tendon stem cells and M1 macrophages, thereby counteracting aging-related repair deficits (67). Thus, targeting the temporal dynamics of macrophage polarization and restoring the M1-to-M2 transition represent promising strategies for treating diabetic wounds and aging-impaired healing.

Chronic diseases often involve pathological shifts in macrophage polarization. In liver fibrosis Warburg effect-like metabolic reprogramming drives macrophages toward a pro-fibrotic phenotype activating hepatic stellate cells through endoplasmic reticulum stress (ERS) and ferroptosis (68). In aged kidneys PCBP1 downregulation disrupts macrophage iron homeostasis inducing mitochondrial damage and ferroptosis while promoting renal fibrosis via STAT1-mediated epithelial-mesenchymal transition (69). During atherosclerosis LRG1 induces M1 polarization by activating ERK1/2 and JNK pathways exacerbating plaque inflammation (70). For autoimmune diseases PCAF alleviates collagen-induced arthritis by coordinately inhibiting NF-κB and H3K9ac (71) with similar polarization imbalances observed in systemic lupus erythematosus and immune thrombocytopenia (72). In estrogen-related disorders the endothelial SHP2/RIPK1/AP1 axis persistently activates macrophages forming an inflammatory circuit that promotes endometrial hyperplasia (73). Conversely in chronic rhinosinusitis SIRT5 enhances glutamine metabolism to facilitate M2 polarization worsening edema (74). For chronic obstructive pulmonary disease targeting immunoproteasomes such as with ONX-0914 nanoparticles simultaneously suppresses both M1 and M2 polarization alleviating emphysema (75). However, in viral myocarditis, ONX 0914 not only fails to exert anti-inflammatory effects, but also weakens the host’s ability to control the virus by non-selectively disrupting the function of the cardiac proteasome, ultimately exacerbating the cardiac inflammation of viral myocarditis (76). In chronic inflammatory conditions, the level of hepcidin produced by liver cells increases, leading to the internalization and degradation of ferroportin, an iron export protein on the macrophage membrane. This “iron retention” effect locks iron inside macrophages, which helps limit pathogen growth but also leads to a lack of circulating iron used for red blood cell production, which is the core pathological mechanism of Anemia of Inflammation (10, 62). Thus, pathological macrophage polarization represents a common mechanism across chronic diseases and targeting their metabolic reprogramming and signaling pathways holds significant therapeutic potential.

As a central hub of immune regulation, macrophage polarization coordinates tissue repair and immune defense through temporal phenotypic switching, while its dysregulation impairs healing and drives chronic diseases. Smart material strategies targeting metabolic reprogramming, key signaling pathways such as JAK-STAT and ERK, and the temporal dynamics of polarization offer novel approaches to enhance tissue regeneration and treat chronic inflammatory conditions.

### 1.4 Objective and scope of the review

Macrophage polarization stands as a central process in immune regulation, and a deeper understanding of its molecular mechanisms and disease relevance holds significant importance for biomedical research. This review systematically integrates recent advances in the field to provide a comprehensive knowledge framework. We first decipher the molecular regulatory networks governing macrophage polarization, including classical signaling pathways such as TLR/NF-κB, STAT family proteins, and PPARγ, which direct M1/M2 phenotypic commitment, while also emphasizing the spectrum-like nature of polarization beyond the traditional dichotomy. Furthermore, we highlight the synergistic roles of epigenetic modifications and metabolic reprogramming in mediating phenotypic plasticity. Next, we systematically link polarization states with pathological processes across diseases, detailing the dual roles of macrophages in chronic inflammation, fibrosis, tumor immune microenvironments, metabolic disorders, and neurodegenerative diseases; we analyze both the mechanisms by which M1 macrophages mediate tissue damage and the contributions of M2 macrophages to dysregulated tissue repair. In terms of translational medicine, this review evaluates the therapeutic potential of targeting polarization strategies, covering small molecule drugs, metabolic modulators, immunotherapy, as well as reprogramming techniques based on exosomes or gene editing, and discusses major challenges in clinical translation, such as microenvironment dependency, limitations in delivery systems, and the lack of biomarkers. Finally, by outlining the potential of emerging technologies like single-cell spatial omics, computational modeling, and synthetic biology in deciphering polarization heterogeneity, this review aims to provide a theoretical foundation for developing individualized macrophage-targeted therapies and to facilitate the translation from basic mechanistic research to clinical intervention.

## 2 The core function of macrophages

As pivotal components of the innate immune system, macrophages play an indispensable role in maintaining tissue homeostasis, eliminating pathogens and aberrant cells, and initiating and modulating adaptive immunity through their potent phagocytic capacity, precise efferocytosis, and professional antigen presentation capabilities.

### 2.1 Phagocytosis

Phagocytosis serves as a fundamental mechanism by which macrophages clear pathogens, apoptotic cells, and abnormal targets such as cancer cells. Within the tumor microenvironment (TME), macrophage phagocytic activity is finely regulated, and targeting “don’t eat me” signals (e.g., CD47) or enhancing “eat me” signals has emerged as a crucial immunotherapeutic strategy (77, 78). For instance, in B-cell lymphoma, inhibition of the pentose phosphate pathway (PPP) metabolically reprograms macrophages via the UDPG-Stat1-Irg1-itaconate axis, thereby enhancing phagocytic clearance of lymphoma cells (79). Similarly, blocking the interaction between tumor cell-secreted vitronectin (Vtn) and its receptor C1qbp on macrophages effectively counteracts the “don’t eat me” signal, augmenting tumor cell phagocytosis and suppressing triple-negative breast cancer progression (80). Furthermore, engineered approaches, such as CAR-macrophages targeting HER2 and CD47, have demonstrated efficacy in promoting phagocytosis of ovarian cancer cells and activating adaptive immunity (81). During infections, macrophages constitute the first line of defense against pathogens like Staphylococcus aureus, although their phagocytic function is often subverted through diverse evasion mechanisms employed by the pathogens (82). Thus, targeted modulation of phagocytosis represents a promising frontier in cancer immunotherapy and host defense.

### 2.2 Efferocytosis

Efferocytosis refers to the specific recognition and clearance of apoptotic cells by macrophages, a process vital for inflammation resolution and tissue repair. Efficient efferocytosis not only prevents secondary necrosis and inflammation caused by the leakage of apoptotic cell contents but also actively induces macrophages to secrete anti-inflammatory factors like IL-10, thereby promoting tissue repair (83). In atherosclerosis, defective efferocytosis leads to the accumulation of apoptotic cells within plaques, exacerbating necrotic core formation and inflammation; conversely, modulating this process, such as through a fish oil-rich diet, can ameliorate disease progression (84). In metabolic dysfunction-associated steatohepatitis (MASH), impaired TIM4 receptor-mediated efferocytosis by liver macrophages (e.g., Kupffer cells) results in failed clearance of apoptotic hepatocytes, thereby driving liver fibrosis progression (85). Mechanistically, efferocytosis can induce macrophage proliferation, a process reliant on apoptotic cell-derived nucleotides activating the mTORC2/Rictor pathway and efferocytosis-generated lactate stabilizing the Myc protein via GPR132 signaling, collectively expanding the pro-resolving macrophage pool and facilitating tissue injury resolution (86, 87). Additionally, the Sigma-1 receptor regulates macrophage efferocytic function by activating Rac1, conferring neuroprotection in cerebral ischemia-reperfusion injury (88). Therefore, efferocytosis is not merely a clean-up process but an active driver of inflammation resolution and tissue healing.

### 2.3 Antigen presentation

As professional antigen-presenting cells (APCs), macrophages bridge innate and adaptive immunity by processing and presenting antigenic peptides to T cells via major histocompatibility complex (MHC) molecules. In lung cancer, alveolar macrophages can support the expansion of CD8^+^ memory T cells and promote the generation of CD103^+^ CD8^+^ tissue-resident memory T cells through antigen cross-presentation, which is crucial for protection against reinfection with respiratory viruses like influenza (89). However, within the TME, the antigen-presenting function of tumor-associated macrophages (TAMs) is often suppressed or co-opted, thereby driving T cell exhaustion. Studies in glioblastoma have shown that persistent antigen presentation by TAMs themselves, rather than by tumor cells, promotes the transition of T cells from a progenitor exhausted state to a terminally exhausted state; depleting TAMs reverses this process and enhances the efficacy of immune checkpoint blockade (78). This suppression involves specific signaling pathways; for example, AMPK-dependent Parkin activation downregulates MHC-I expression on macrophages via an autophagy-dependent pathway, thereby inhibiting antigen presentation and promoting tumor progression (90). Moreover, in cervical cancer, CD8^+^ T cell-derived IFN-γ upregulates HK3 expression in TAMs via the STAT1 axis. HK3, in turn, impairs TAM cross-presentation capacity by promoting TFEB nuclear translocation and excessive lysosomal activation and antigen degradation, thereby undermining anti-tumor immunity; targeting HK3 restores antigen presentation and synergizes with immune checkpoint blockade (91). Consequently, harnessing or rescuing macrophage antigen presentation is a critical strategy for reinvigorating anti-tumor immunity.

In summary, phagocytosis, efferocytosis, and antigen presentation constitute the three core functional pillars enabling macrophages to perform immune surveillance and maintain homeostasis. These functions are not isolated but are interconnected and synergistic, collectively determining the direction and outcome of immune responses. A deeper understanding of their regulatory mechanisms is paramount for developing novel immunotherapeutic strategies against a wide spectrum of diseases, including infections, cancer, autoimmune disorders, and fibrotic conditions.

## 3 Molecular mechanisms of macrophage polarization

Macrophage functional plasticity is central to immune defense tissue homeostasis and repair processes primarily manifested through the dynamic balance between pro-inflammatory M1 and anti-inflammatory reparative M2 phenotypes. This process is precisely regulated by multi-layered molecular networks. Key signaling pathways such as TLR/NF-κB JAK/STAT and PPARs receive and integrate microenvironmental signals directly driving macrophage polarization. Epigenetic mechanisms including DNA methylation histone modifications and non-coding RNAs fine-tune gene expression at transcriptional and post-transcriptional levels granting cellular plasticity and long-term memory. Meanwhile metabolic reprogramming provides energy and biosynthetic precursors for polarization—M1 macrophages favor glycolysis and the pentose phosphate pathway while M2 types rely on OXPHOS and FAO. Metabolic intermediates also act as signaling molecules and epigenetic regulators further shaping immune phenotypes. These mechanisms intersect synergize or antagonize collectively forming a sophisticated regulatory network for polarization.

### 3.1 Key signaling pathways

**Figure 1:**
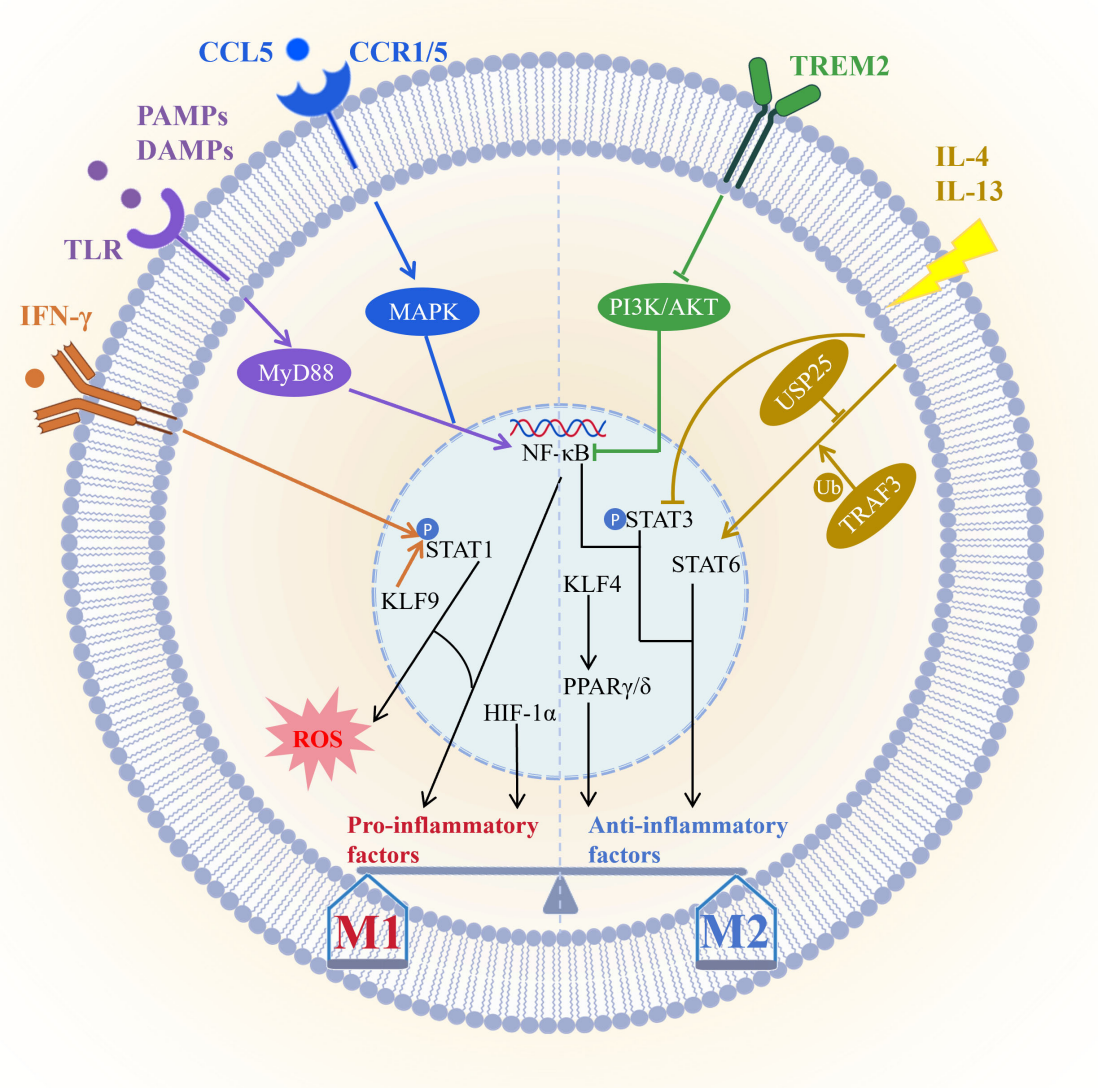
Core signaling pathways governing macrophage polarization. M1 polarization is primarily driven by the TLR/NF-κB and IFN-γ/STAT1 pathways, and further modulated by factors such as KLF9, the CCL5/CCR1/CCR5–MAPK/NF-κB axis, and HIF-1α; M2 polarization, conversely, relies on the IL-4/IL-13/STAT6 pathway—regulated by USP25 and TRAF3—and PPARs, which are activated by KLF4, with TREM2 promoting M2 transition via suppression of the PI3K/AKT/NF-κB pathway. These signaling networks dynamically regulate the M1/M2 balance through synergistic or antagonistic interactions, thereby influencing inflammatory responses, tissue repair, and disease progression. Picture created using BioRender and BioGDP.

The molecular mechanisms of macrophage polarization depend on a complex signaling network which centers on maintaining the dynamic balance between the pro-inflammatory M1 phenotype and the anti-inflammatory reparative M2 phenotype (**Figure 1**). M1 polarization is primarily driven by Toll-like receptor signaling pathways especially through the TLR/NF-κB axis. Pathogen or damage-associated molecular patterns activate MyD88-dependent signaling via TLRs leading to NF-κB activation and induction of pro-inflammatory cytokines. For instance hippuric acid enhances M1 polarization through the TLR/MyD88 pathway (92), whereas VP-NP nanoparticles alleviate colitis by suppressing endosomal TLR signaling (93). IFN-γ serves as a key initiating signal for M1 polarization by binding to IFNGR receptors and activating the JAK/STAT1 pathway resulting in STAT1 phosphorylation and upregulation of effector molecules such as iNOS CD86 and ROS (94, 95). KLF9 promotes macrophage recruitment and differentiation by modulating the IFN-γ/STAT1 axis contributing to post-infarction repair (96). Furthermore, CCL5 directly promotes M1 polarization and suppresses M2 polarization via CCR1/CCR5-mediated activation of MAPK and NF-κB pathways (97). However, CCL5, as a downstream effector molecule of GZMK, interacts with it and activates the ERK signaling pathway, thereby driving the proliferation, migration, and invasion of synovial fibroblasts, and inhibiting their apoptosis and ferroptosis, ultimately accelerating the progression of experimental rheumatoid arthritis (98). Under hypoxic conditions HIF-1α stability increases promoting glycolysis and IL-1β release however in sepsis FGF21 suppresses M1 activation by promoting autophagic degradation of HIF-1α (99), while in atherosclerosis, pyruvate carboxylase exacerbate inflammation via HIF-1α (100). In summary M1 polarization is finely regulated by multiple pathways significantly influencing the progression and intensity of inflammatory responses.

M2 polarization is primarily driven by IL-4 and IL-13 through activation of the STAT6 signaling pathway and induces the expression of anti-inflammatory genes such as Arg1 (101). The deubiquitinating enzyme USP25 promotes M2 polarization by stabilizing STAT6 (102), while TRAF3 plays a key role by regulating STAT6 ubiquitination (103). PPARγ and PPARδ act as lipid metabolic sensors that enhance FAO and anti-inflammatory factor production and are also essential for M2 polarization (101). KLF4 is highly expressed in M2 macrophages and serves as a core regulator of polarization; low-dose decitabine enhances KLF4 binding to the PPARγ promoter and promotes M2 polarization (104, 105). However, the specific knockout of KLF4 in AdvSca1-SM cells induces it to differentiate into a more protective cell phenotype, and enhances plaque stability by changing plaque composition (such as reducing necrotic core and increasing fiber cap thickness), thus inhibiting atherosclerosis from developing toward instability (106). PPARγ agonists such as pioglitazone can reprogram macrophage metabolic states (107). Conversely, exosomal miR-9 from HPV-positive head and neck squamous cell carcinoma suppresses PPARδ and induces an M1 phenotype (108). Additionally, IL-13 promotes microglia/macrophage polarization toward the M2 type by inhibiting STAT3 phosphorylation (109). TREM2 facilitates the transition from M1 to M2 macrophages by suppressing PI3K/AKT and NF-κB signaling and downregulating CXCL3, thereby alleviating osteoarthritis (110). Together these pathways shape the anti-inflammatory, reparative, and tissue-homeostatic functions of M2 macrophages.

Macrophage polarization involves multilayered crosstalk among signaling pathways. For example, PPARγ dephosphorylation at T166 enhances lipid synthesis and promotes reparative factor expression via STAT3 (111). Meanwhile, HIF-1α forms a positive feedback loop with glycolysis to sustain the M1 inflammatory phenotype. Immune checkpoints also participate in this process. IL-4/STAT6 signaling induces FcγRIIB expression, which mediates resistance to PD-1 antibody therapy (112). Furthermore, its downstream effector Siglec-10 acts on HIF-1α to foster an immunosuppressive microenvironment (113). Interventions targeting these nodal points—such as nanodrugs modulating TLR/STAT signaling or metabolites regulating HIF-1α activity—offer novel therapeutic avenues. Thus, the balance of macrophage polarization is coordinately regulated by STAT, NF-κB, PPAR, and other pathways (**Figure 1**). This highly plastic network not only dictates immune homeostasis and disease outcomes but also provides a theoretical foundation for macrophage-targeted therapies, including nanomedicine and metabolic interventions.

### 3.2 Epigenetic regulation

**Figure 2:**
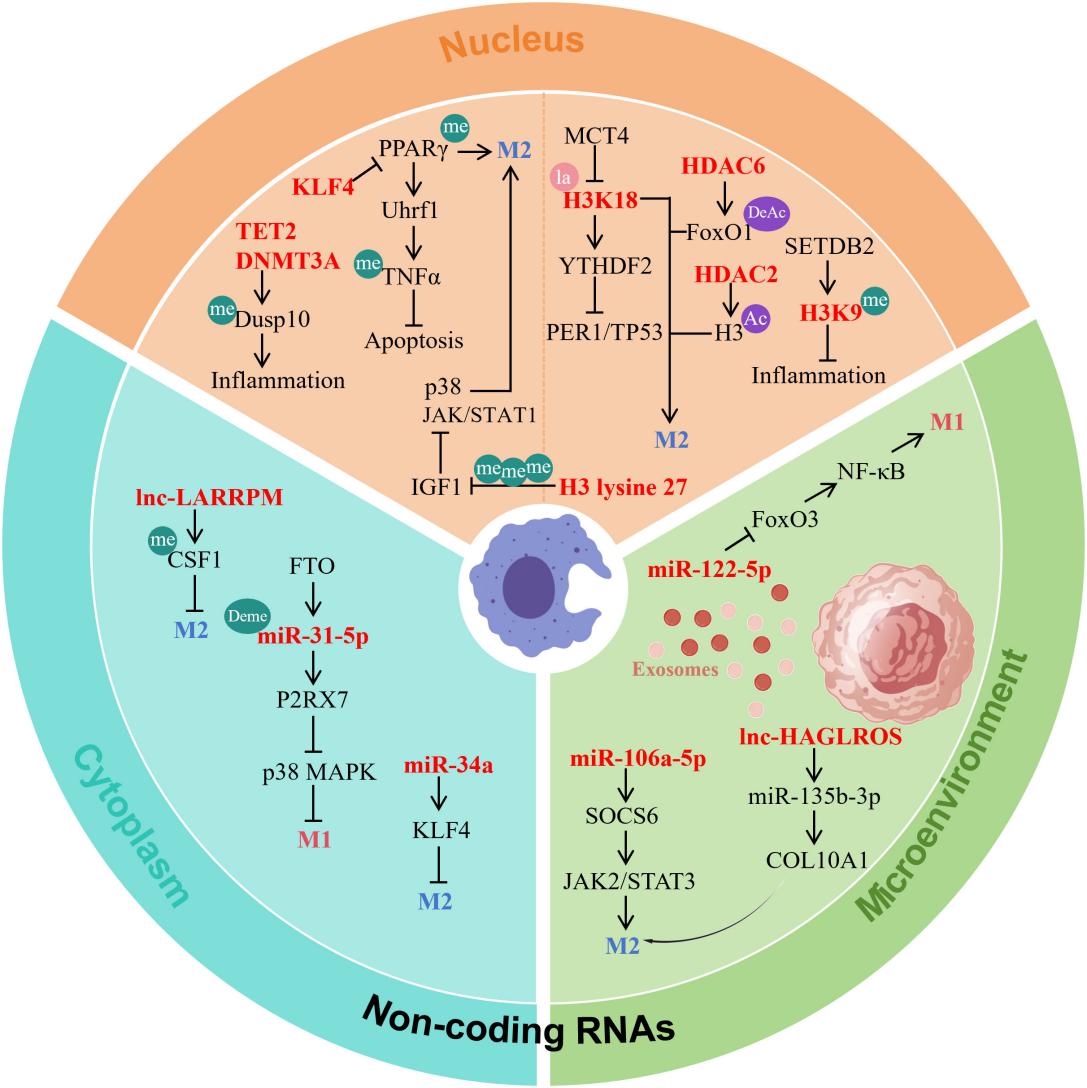
Macrophage polarization is precisely regulated by multi-layered epigenetic mechanisms. (1) DNA methylation: Dysregulation of TET2/DNMT3A alters the methylation status of key gene promoters such as Dusp10 and IGF1, thereby modulating inflammatory signaling pathways; KLF4 promotes M2 polarization by reducing PPARγ promoter methylation; Uhrf1-mediated hypermethylation of the TNF promoter suppresses cell death. (2) Histone modifications: Lactate accumulation induces H3K18 lactylation, which facilitates repair gene expression and drives M2 polarization; HDAC6 and HDAC2 regulate polarization by modulating FoxO1 acetylation and H3 deacetylation; SETDB2-mediated H3K9 methylation is critical for silencing inflammatory genes. (3) Non-coding RNAs: lncRNAs such as LARRPM inhibit M2 polarization by enhancing CSF1 methylation; miRNAs (e.g., miR-34a) and FTO-mediated m6A demethylation modifications (e.g., miR-31-5p) target KLF4 or P2RX7, affecting their stability and function; exosomal ncRNAs (including miR-106a-5p, lncRNA HAGLROS, and miR-122a-5p) regulate polarization by targeting signaling molecules such as SOCS6 or FoxO3. These three mechanisms act synergistically to precisely control the plasticity and polarization state of macrophages. Me, methylation; Deme, demethylation; la, lactylation; Ac, acetylation; DeAc, deacetylation. Picture created using BioGDP.

Macrophage polarization is epigenetically regulated through DNA methylation, histone modifications, and non-coding RNAs, which collectively fine-tune gene expression and influence macrophage plasticity and disease roles (**Figure 2**). Loss of TET2 elevates methylation in the Dusp10 promoter, thereby suppressing its expression and enhancing JNK phosphorylation. This promotes BRCC3-mediated NLRP3 deubiquitination and inflammasome activation, ultimately accelerating atherosclerosis (114). Impaired serine metabolism reduces H3K27 trimethylation on histones, which upregulates IGF1 and activates the p38–JAK/STAT1 pathway to drive M1 polarization while inhibiting M2 polarization (115). Loss-of-function mutations in DNMT3A cooperate with TET2 to alter DNA methylation patterns, amplifying inflammatory phenotypes in macrophages and worsening vascular pathology (116). In contrast, low-dose decitabine reduces methylation at the KLF4 and PPARγ promoters, promoting their binding and activating anti-inflammatory gene expression to induce M2 polarization (105). In inflammatory microenvironments, Uhrf1-dependent hypermethylation of the TNF-α promoter inhibits macrophage necroptosis, a process mediated by PPARγ-regulated Uhrf1 transcription (117). Collectively, DNA methylation precisely controls key gene expression, significantly affecting macrophage polarization and inflammatory responses, making it a potential epigenetic therapeutic target in diseases such as atherosclerosis.

Histone modifications dynamically regulate macrophage polarization with lactylation emerging as a prominent mechanism at the metabolism-epigenetics interface. MCT4 deficiency induces lactate accumulation which drives H3K18la modification to activate repair genes and promote M2 polarization thereby suppressing atherosclerosis (118). In the TME histone lactylation upregulates YTHDF2 which promotes tumorigenesis by degrading PER1/TP53 mRNA (119). Acetylation modifications equally contribute to polarization dynamics. HDAC6 mediates FoxO1 deacetylation and enhances its phosphorylation promoting M2 polarization to ameliorate periodontitis (120). Meanwhile the HDAC2/SP1 axis remodel H3 acetylation to foster M2-like tumor-associated macrophage polarization (121). SETDB2 deficiency reduces H3K9 methylation impairing gene silencing and exacerbating inflammatory responses (122). Thus, histone lactylation acetylation and methylation collectively fine-tune gene expression profiles significantly influence macrophage polarization and serve as pivotal epigenetic nodes integrating metabolism inflammation and disease progression.

Non-coding RNAs regulate macrophage phenotype switching through post-transcriptional mechanisms. The lncRNA LARRPM recruits TET1 to the LINC00240 promoter inducing its demethylation and suppresses tumor progression. It also reduces TET1 binding to the CSF1 promoter promoting methylation and thus inhibiting M2 polarization (123). Exosomal ncRNAs play important roles in intercellular communication. Colorectal cancer cell-derived miR-106a-5p drives M2 polarization by targeting SOCS6 and activating the JAK2/STAT3 pathway (124). Breast cancer-derived exosomal lncRNA HAGLROS promotes M2 conversion through the miR-135b-3p/COL10A1 axis (125). miR-34a inhibits M2 polarization by suppressing KLF4 (126). Additionally chemical modifications of miRNAs regulate their functions. FTO-mediated m6A demethylation of miR-31-5p enhances its stability and inhibits the p38 MAPK pathway by targeting P2RX7 thereby blocking M1 polarization (127). Plasma exosomal miR-122-5p from systemic lupus erythematosus patients promotes M1 polarization and worsens nephritis by suppressing FoxO3 and activating NF-κB (128). Thus, lncRNAs exosomal ncRNAs and modified miRNAs form a multi-level regulatory network that precisely controls macrophage polarization making them crucial regulatory factors and therapeutic targets in inflammation and cancer.

In summary, the epigenetic regulatory network governing macrophage polarization dynamically modulates their plasticity through coordinated actions of three primary mechanisms, including DNA methylation, histone modifications such as lactylation, acetylation and methylation, as well as non-coding RNAs comprising lncRNAs, exosomal ncRNAs and modified miRNAs. These mechanisms profoundly influence the expression of key genes, thereby critically contributing to the progression of atherosclerosis, cancer and inflammatory diseases. This intricate interplay offers a theoretical foundation and therapeutic opportunities for novel treatment strategies targeting the metabolic-epigenetic cross-regulation.

### 3.3 Metabolic reprogramming

**Figure 3:**
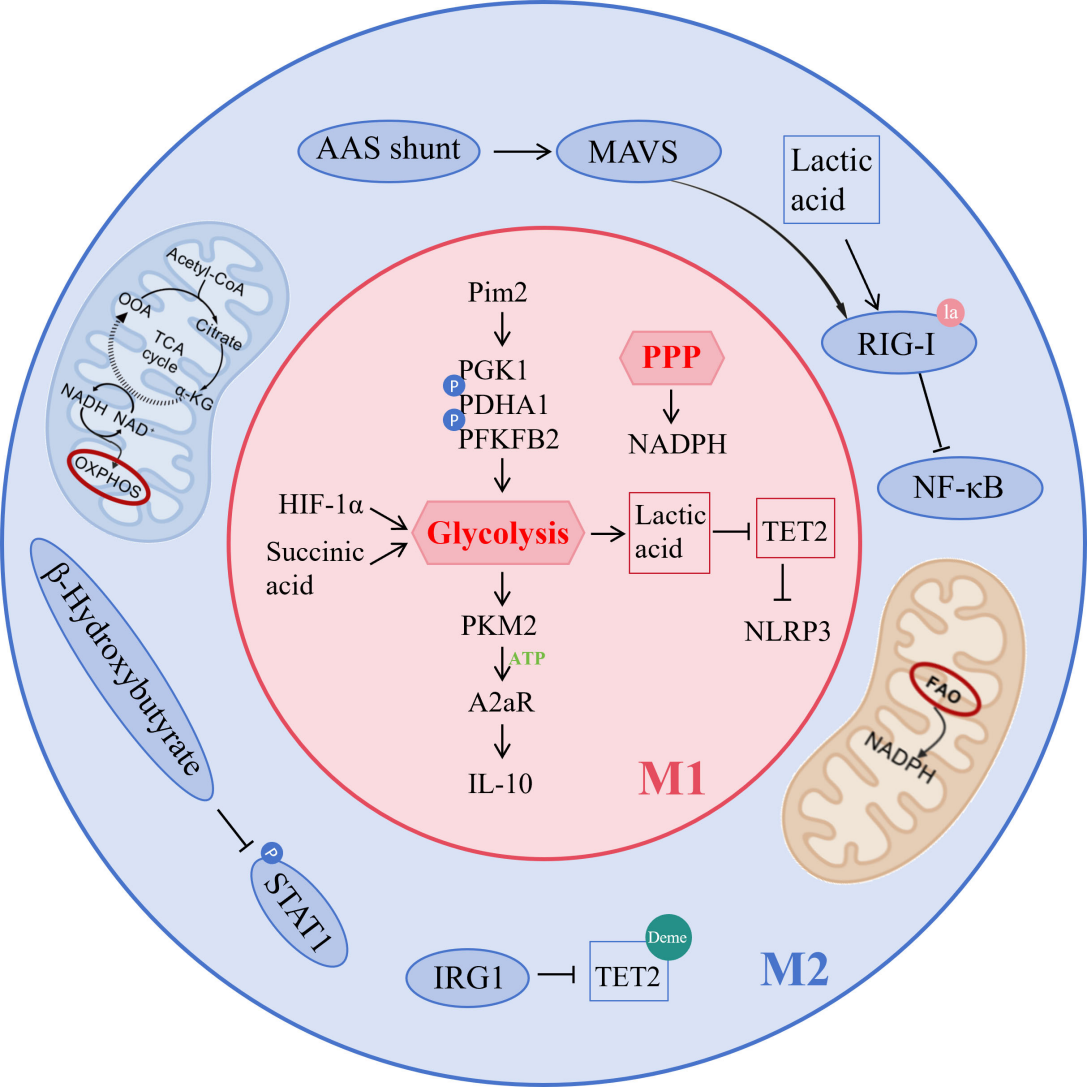
Core genes and pathway networks in metabolic reprogramming during macrophage polarization. M1 polarization is predominantly driven by glycolysis and the pentose phosphate pathway (PPP), which are precisely regulated by multiple factors including HIF-1α, PKM2 (via modulation of A2aR signaling), lactate, succinate, and Pim2 (phosphorylating glycolytic enzymes PGK1, PDHA1, and PFKFB2), and further feedback-activates the NLRP3 inflammasome. In contrast, M2 polarization primarily relies on FAO and OXPHOS, regulated by key molecules such as IRG1 (suppressing TET2), lactate (inhibiting NF-κB via RIG-I lactylation), β-hydroxybutyrate (mediating STAT1 modification), and the aspartate-argininosuccinate shunt (activating RIG-I through MAVS succinylation). Specific metabolites generated through metabolic reprogramming dynamically shape macrophage polarization toward pro-inflammatory (M1) or anti-inflammatory repair (M2) phenotypes by modulating enzymatic activities (e.g., TET2) and signaling pathways (including A2aR, NLRP3, NF-κB, and STAT1). Picture created using BioRender.

Metabolic reprogramming during macrophage polarization serves as a core driver of functional plasticity. M1 pro-inflammatory polarization primarily relies on glycolysis and the pentose phosphate pathway (PPP), whereas M2 anti-inflammatory and reparative polarization favors OXPHOS and FAO. Intermediates from these metabolic pathways not only supply energy and biosynthetic precursors but also profoundly shape the immune phenotype of macrophages through signaling and epigenetic mechanisms (**Figure 3**). In M1 polarization, stimuli such as lipopolysaccharide enhance glycolytic flux by stabilizing HIF-1α and promote tetramerization of pyruvate kinase M2 (PKM2). Released ATP is converted to adenosine via ectonucleotidases, activating adenosine receptor A2a (A2aR) and inducing IL-10 production (129). The regulatory effect of lactate on the NLRP3 inflammasome exhibits a significant “double-edged sword” characteristic, meaning that in different pathological contexts, lactate can either promote or inhibit NLRP3 activation through distinct molecular mechanisms. On one hand, lactate and the protein lactylation it mediates have been found to significantly suppress NLRP3 inflammasome activation. For instance, in an acute pancreatitis model, lactate derived from *Bifidobacterium* inhibited macrophage-associated pancreatic and systemic inflammatory responses in a TLR4/MyD88- and NLRP3/Caspase-1-dependent manner (130). In macrophages from G6PT-deficient patients, lactate accumulation induces lactylation at the histone H3K18 site, upregulating the expression of ALKBH5. This enzyme, in turn, reduces the m6A modification level of NLRP3 mRNA, thereby destabilizing it and ultimately inhibiting NLRP3 inflammasome activation (131). On the other hand, under specific disease conditions, enhanced lactate metabolism and lactylation can drive NLRP3 activation. In systemic lupus erythematosus (SLE) pregnancy, neutrophil extracellular traps (NETs) promote glycolysis and lactate production in trophoblasts, leading to NLRP3 lactylation, which subsequently activates the inflammasome and triggers pyroptosis (132). In a perivascular adipose tissue inflammation model, macrophages deficient in SIRT3 exhibited a metabolic switch from oxidative phosphorylation to glycolysis, resulting in lactate accumulation and subsequent promotion of NLRP3 inflammasome activation and IL-1β secretion (133).In summary, the regulation of the NLRP3 inflammasome by lactate is not unidirectional. Its ultimate effect is highly dependent on the cell type, disease model, and specific microenvironmental signals. Furthermore, succinate delivered via tumor-derived microvesicles triggers succinylation of isocitrate dehydrogenase 2 (IDH2) and histone H3K122, thereby enhancing lactate dehydrogenase A (LDHA) expression and forming a positive feedback loop that promotes glycolysis (134). Such metabolic reprogramming engages in complex crosstalk with inflammatory signaling pathways. For example, Pim2 kinase directly activates glycolysis by phosphorylating glycolytic enzymes PGK1, PDHA1, and PFKFB2, driving M1 polarization in inflammatory arthritis (135). Glycolytic reprogramming itself can further activate the NLRP3 inflammasome, exacerbating acute lung injury and respiratory distress syndrome (136). Thus, metabolic reprogramming not only furnishes macrophages with energy and biosynthetic precursors but also acts as a central mechanism fine-tuning their immunophenotype and functional state.

M2 polarization is characterized by enhanced mitochondrial metabolism, primarily relying on FAO and OXPHOS for energy production. The key anti-inflammatory metabolite itaconate, synthesized via IRG1, not only inhibits TET2-mediated DNA demethylation to alleviate bone destruction in rheumatoid arthritis (137), but also can be delivered locally through functionalized implants to suppress M1 polarization and promote vascular endothelial repair (138). Citrate exerts dual roles in bone homeostasis exogenous citrate-based scaffolds redirect metabolic flux toward OXPHOS by inhibiting glycolytic enzymes, thereby promoting M2 polarization and improving osteoporosis (139). Lactate drives M2 polarization via RIG-I lactylation to inhibit the NF-κB pathway (140). Recent studies have shown that β-hydroxybutyrate can induce lysine β-hydroxybutyrylation on STAT1 (141). This emerging post-translational modification has been demonstrated to suppress LPS-induced STAT1 phosphorylation and its downstream M1-type gene expression, offering a new perspective on how metabolites regulate immune responses. However, whether this mechanism holds true universally across all macrophage contexts still requires further validation through additional research. Furthermore, reprogramming of the urea and TCA cycles forms the aspartate-arginosuccinate shunt (AAS), generating fumarate during antiviral immunity that ultimately activates the RIG-I-like receptor pathway through succination of the MAVS protein (59). Thus, specific metabolites and their regulatory pathways in M2 macrophages collectively constitute a critical hub for immunomodulation and tissue repair.

In the tumor microenvironment, cancer cells secrete arginine to remodel the metabolic landscape of tumor-associated macrophages (TAMs), thereby driving their polarization toward a pro-tumor phenotype. Mechanistically, polyamines derived from arginine metabolism promote DNA hypomethylation via thymine DNA glycosylase (TDG), enhancing the immunosuppressive function of TAMs and facilitating malignant progression in breast cancer (142). Arginine metabolism exhibits dynamic plasticity, and its flux can be monitored in real time using novel fluorescent sensors; it also plays a regulatory role in key processes such as macrophage activation, phagocytosis, and senescence (143). In inflammatory bone destruction, L-arginine reprograms osteoclast energy metabolism by shifting their metabolic profile from glycolysis toward oxidative phosphorylation and perturbing purine metabolism, ultimately suppressing arthritis and bone resorption (144). Sphingolipid metabolism also undergoes systematic rearrangement upon TLR4 activation, with newly synthesized species such as ceramide playing stage-specific regulatory roles during both pro-inflammatory and resolution phases in macrophages (145). Within the tryptophan metabolic axis, efferocytosis drives an IDO1-dependent tryptophan-kynurenine metabolic flux in macrophages, activating the aryl hydrocarbon receptor (AhR) to promote inflammation resolution and tissue repair (146). Conversely, microbiota-derived tryptophan metabolites, such as indole, can also induce an immunosuppressive phenotype in TAMs via AhR signaling, thereby impairing anti-tumor T cell function (147). Beyond amino acid metabolism, the one-carbon metabolic enzyme MTHFD2 exhibits non-canonical functions by directly binding and inhibiting PTEN, thereby modulating Akt pathway activity and fine-tuning the M1/M2 polarization balance (148). The key mitochondrial dynamics protein Drp1 interacts with hexokinase 1 (HK1) to induce mitochondrial permeability transition pore (mPTP) opening, leading to NLRP3 inflammasome activation and exacerbation of localized inflammation in periodontitis (149). Meanwhile, mitochondrial arginase-2 (Arg2) acts as a central executor of IL-10-mediated metabolic reprogramming. By enhancing succinate dehydrogenase (Complex II) activity to promote oxidative phosphorylation and cooperatively suppressing HIF-1α and IL-1β expression, Arg2 precisely regulates the inflammation resolution process (150).In summary, macrophage metabolic reprogramming extends far beyond a simple switch in energy supply, representing instead a highly integrated and cross-pathway global regulatory network. From amino acid metabolism—such as arginine and tryptophan—to sphingolipid and one-carbon metabolism, and further to the fine-tuning of mitochondrial dynamics and respiratory chain function, the metabolic network profoundly determines macrophage polarization fate and functional output by influencing epigenetic states, inflammasome activation, cytokine expression, and immune checkpoint signaling. Understanding this multilayered and dynamically evolving metabolic dialogue not only reveals the complex nature of immunometabolic regulation but also offers novel perspectives and potential therapeutic targets for diseases through targeting macrophage metabolism.

In summary, metabolic reprogramming during macrophage polarization precisely shapes immune function through distinct energy metabolism patterns and the signaling and epigenetic regulation mediated by metabolic intermediates. These metabolic networks not only form cross-regulatory feedback loops with inflammatory signaling, contributing to disease pathogenesis and progression, but also enable immune phenotype reprogramming via targeting metabolic enzymes, thus offering novel therapeutic strategies for cancer immunity, tissue repair, and immune-related diseases.

## 4 Disease implications of macrophage polarization

**Figure 4:**
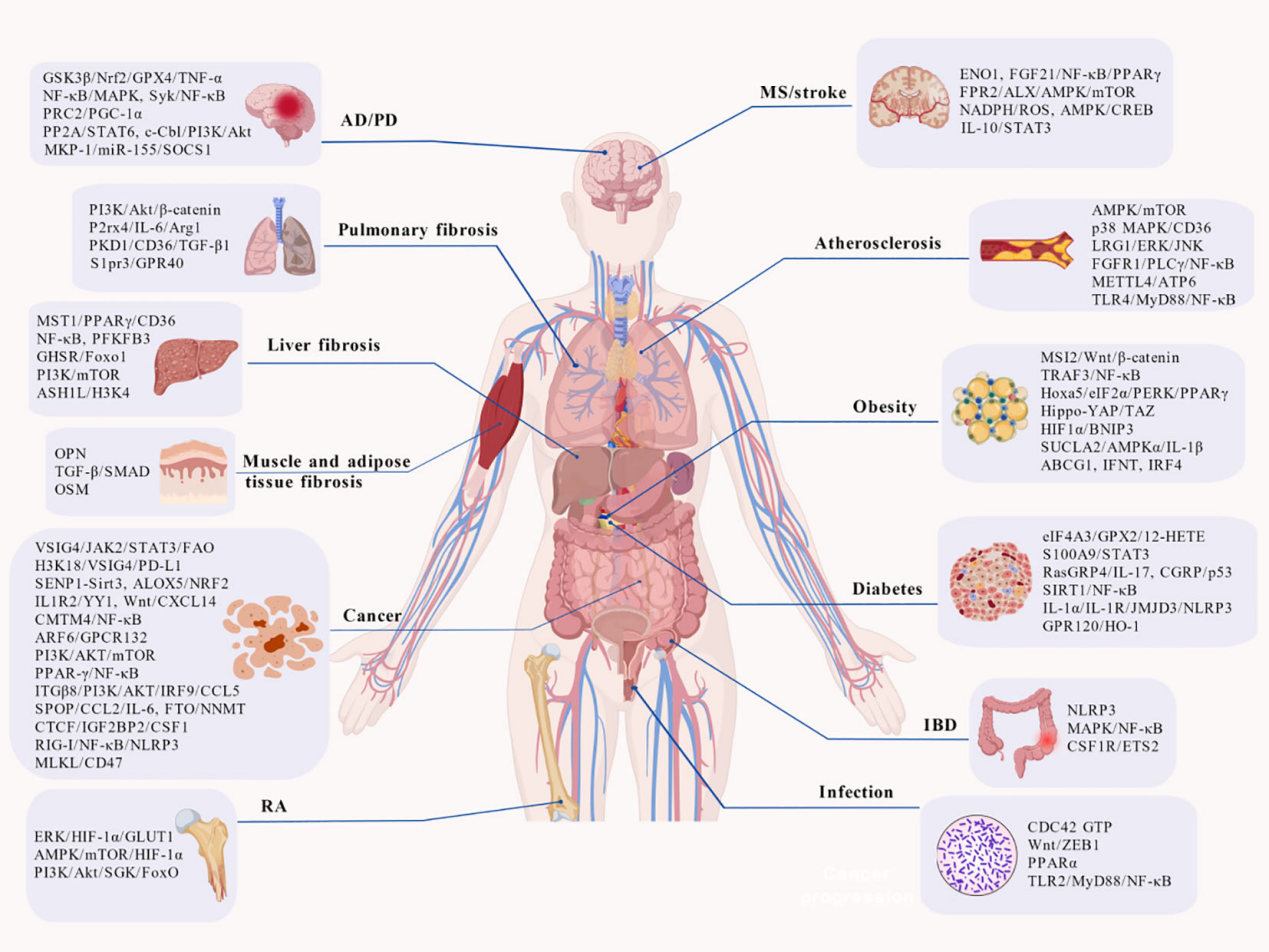
Dysregulated macrophage polarization serves as a critical mechanism driving the progression of various diseases. This figure summarizes key signaling pathways associated with macrophage polarization in the following five categories of pathologies: (1) inflammatory and autoimmune diseases, such as rheumatoid arthritis, atherosclerosis, and inflammatory bowel disease; (2) fibrotic disorders, including hepatic fibrosis, pulmonary, muscle and adipose tissue fibrosis; (3) cancer, involving the immunosuppressive functions of TAMs and their roles in tumor progression and metastasis; (4) metabolic diseases, such as obesity-induced adipose tissue inflammation, diabetes, and its complications; (5) neurodegenerative disorders, including Alzheimer’s disease, Parkinson’s disease, multiple sclerosis, and stroke; and (6) infection. Picture created using BioGDP.

The dynamic imbalance in macrophage polarization states serves as a central driver in the pathogenesis of various diseases. Specifically, dysregulated phenotypic switching between pro-inflammatory (M1) and anti-inflammatory reparative (M2) macrophages within the diseased microenvironment profoundly influences key processes such as inflammatory progression, tissue repair, and fibrotic remodeling. This regulation occurs through immune response modulation, metabolic reprogramming, and intercellular interactions. This review systematically examines the critical roles of macrophage polarization across several major disease domains. These include inflammatory and autoimmune diseases such as rheumatoid arthritis, atherosclerosis, and inflammatory bowel disease, fibrotic disorders like hepatic, pulmonary, muscle and adipose tissue fibrosis, cancer particularly through TAMs which mediate immunosuppression and facilitate tumor progression and metastasis, metabolic diseases including obesity-associated adipose tissue inflammation, diabetes and its complications, and neurodegenerative conditions such as Alzheimer’s disease, Parkinson’s disease, multiple sclerosis, and stroke, as well as infection (**Figure 4**). Finally, this review delves into the clinical translational potential of targeting macrophage polarization for therapeutic intervention.

### 4.1 Inflammatory and autoimmune diseases

The polarization imbalance of macrophages is a common pathological feature of many inflammatory and autoimmune diseases.

#### 4.1.1 Rheumatoid arthritis

Excessive infiltration of pro-inflammatory M1 macrophages into synovial tissue drives chronic inflammation and joint destruction, making their conversion to anti-inflammatory M2 phenotypes a key therapeutic objective. Nanotechnology-based drug delivery systems enable precise targeting of articular lesions and significantly improve treatment efficacy. For instance, icariin-loaded adipose-derived stem cell exosomes accumulate in joint cavities and facilitate the shift from M1 to M2 polarization by suppressing the ERK/HIF-1α/GLUT1 pathway, thereby reducing glycolysis and alleviating synovitis and cartilage damage in collagen-induced arthritic rats (151). Similarly, macrophage membrane-biomimetic nanoparticles (2-APB@DGP-MM) achieve localized drug release via matrix metalloproteinase-responsive peptides, directly reprogramming macrophage polarization to mitigate joint inflammation (152). The liver X receptor inverse agonist SR9243 activates the AMPK/mTOR/HIF-1α axis, inhibiting key glycolytic enzymes such as LDH-A and HK2 in M1 macrophages and markedly reducing bone erosion in adjuvant-induced arthritic rats (153). Meanwhile, bovine serum albumin-bilirubin-platinum nanoparticles (BSA-BR-Pt NPs) scavenge reactive oxygen species and ameliorate hypoxia, switching glycolysis to OXPHOS and consequently reducing pro-inflammatory cytokine secretion in M1 macrophages (154).

Multicellular interaction networks play critical roles in disease progression. Syringin disrupts pathogenic crosstalk between macrophages and fibroblast-like synoviocytes by inhibiting phosphodiesterase 4 (PDE4), thereby reducing inflammatory cytokine release and suppressing abnormal FLS proliferation (155). Engineered M2 macrophage-derived exosome nanocomplexes induce copper-induced cell death in activated T cells. Their fragments are phagocytosed by macrophages, promoting TGF-β secretion and subsequently driving regulatory T cell differentiation to establish antigen-specific immune tolerance (156). Moreover, sinomenine-loaded graphene oxide quantum dot complexes (HA@RFM@GP@SIN NPs) synergistically modulate steroid hormone and amino acid metabolic pathways. They also inhibit the PI3K/Akt/SGK/FoxO signaling cascade in FLS, enabling dual intervention in macrophage polarization and synovial hyperplasia (157). In summary, regulating the metabolic reprogramming of macrophages has become an emerging and effective direction for RA treatment.

#### 4.1.2 Atherosclerosis

Hyperglycemia and dyslipidemia induce Sestrin2 to activate monocytes via the AMPK/mTOR signaling axis, promoting the release of pro-inflammatory factors (M1 phenotype) while suppressing anti-inflammatory factors (M2 phenotype), thereby exacerbating foam cell formation and endothelial adhesion (158). In contrast, transaldolase suppresses the p38 MAPK pathway by maintaining glutathione homeostasis, downregulates the scavenger receptor CD36 expression, and inhibits cholesterol uptake and foam cell formation (159). Epigenetic regulation is also involved in this process, as miR-127-3p targets and inhibits SCD1, reducing unsaturated fatty acid levels and enhancing mitochondrial OXPHOS, which drives macrophage polarization toward the M1 phenotype and promotes plaque progression (160). Meanwhile, exercise-induced lactylation at the MeCP2 K271 site promotes M2 polarization and enhances plaque stability by suppressing RUNX1 transcription (161). Thus, the regulatory network of macrophage polarization represents a critical mechanism in atherosclerosis pathogenesis.

Aberrant activation of inflammatory signaling pathways plays a central role in disease pathogenesis. LRG1 induces M1 macrophage polarization via the ERK/JNK pathway, and its deficiency significantly attenuates atherosclerosis progression (70). Moreover, in macrophages, FGFR1 activates the NF-κB inflammatory cascade through PLCγ, promoting oxidized low-density lipoprotein uptake and lesion formation (162). Mitochondrial dysfunction also contributes to inflammatory regulation. METTL4-mediated 6mA methylation of mitochondrial DNA suppresses ATP6 expression, impairing respiratory chain complex V function and thereby driving macrophage inflammatory responses and accelerating atherosclerosis (163). These findings collectively highlight LRG1, FGFR1, and mitochondrial epigenetic modifications as potential therapeutic targets.

Microenvironmental factors also play critical roles in atherosclerosis. Early intermittent hyperlipidemia accelerates atherosclerotic lesions by disrupting the homeostatic phenotype of LYVE1^+^ macrophages (164). The gut microbiota-derived metabolite indole-3-acetic acid (IAA) promotes M2 polarization and reduces vascular inflammation through suppressing the TLR4/MyD88/NF-κB pathway (165). Additionally, hypercholesterolemia induces depletion of embryo-derived Kupffer cells, which impairs hepatic cholesterol regulation and thereby promotes disease progression (166). It can be seen that macrophage polarization network is a regulation link that cannot be ignored in the process of atherosclerosis.

#### 4.1.3 Inflammatory bowel disease

The mechanosensitive ion channel Piezo1 drives macrophage polarization toward the pro-inflammatory M1 phenotype by activating NLRP3 and NF-κB signaling pathways, thereby promoting colitis development. Specific knockout of Piezo1 in macrophages effectively alleviates chronic intestinal inflammation (167). The traditional Chinese medicine component Polyphyllin VI (PPVI) enhances autophagy activity to suppress NLRP3 inflammasome activation, balances the M1/M2 macrophage ratio, and subsequently reduces intestinal epithelial barrier injury (168). Notably, alcohol intake activates transient receptor potential vanilloid type 1 (TRPV1), enhances calcium influx, activates the MAPK/NF-κB pathway, and induces macrophage polarization toward the pro-inflammatory M2b subtype, thereby aggravating colitis severity (169). Therefore, targeting mechanosensitive channels, autophagy regulation, and environmental sensing pathways may provide multi-dimensional intervention strategies for restoring macrophage homeostasis and suppressing intestinal inflammation.

The regulatory mechanisms governing macrophage differentiation also influence susceptibility to IBD. Colony-stimulating factor 1 receptor signaling promotes the differentiation of monocytes into tolerant intestinal macrophages by downregulating the transcription factor ETS2, while noncoding variants at the ETS2 locus may disrupt this process and thereby increase IBD risk (170). Additionally, dysregulated iron metabolism contributes to pathogenesis, as iron-loaded exosomes released by intestinal epithelial cells are recognized and internalized via macrophage scavenger receptor 1, triggering oxidative stress and inflammatory responses that exacerbate mucosal injury (171). Thus, targeting macrophage differentiation pathways such as the CSF1R/ETS2 axis and iron homeostasis may offer novel molecular interventions and risk stratification strategies for intestinal inflammatory disorders.

### 4.2 Fibrotic disorders

#### 4.2.1 Liver fibrosis

During liver fibrosis progression, macrophage polarization synergistically drives disease development through interactions between metabolic reprogramming and signaling pathways. Chronic liver injury induces metabolic adaptations in macrophages, characterized by aberrant glucose and lipid metabolism along with activation of stress responses such as ERS and autophagy, thereby promoting their transition toward inflammatory and fibrotic phenotypes (68, 172). Specifically, MST1 kinase enhances the PPARγ/CD36 pathway to improve phagocytic function, upregulate fibrolytic genes including Arg1 and Mmps, and suppress the NF-κB signaling pathway, ultimately ameliorating schistosome egg-induced granulomas and liver fibrosis (173). Conversely, the GHSR/Foxo1 axis promotes TGF-β1 secretion in macrophages via PKA-mediated phosphorylation of Foxo1 at S273, activating hepatic stellate cells and exacerbating inflammatory infiltration, thus aggravating carbon tetrachloride-induced liver fibrosis (174). Moreover, the natural flavonoid astilbin modulates the crosstalk between hepatic stellate cells and macrophages by activating the PXR receptor and inhibiting PINK1/Parkin-mediated mitophagy, improving the fibrotic microenvironment (175). Additionally, SGLT2 inhibitors reprogram macrophage metabolism by downregulating the glycolytic enzyme PFKFB3, facilitate the transition from M1 to M2 phenotype, and indirectly inhibit lipid accumulation in hepatocytes (176). Therefore, targeting key nodes within the macrophage metabolic-signaling network—such as PPARγ/CD36, Foxo1 phosphorylation, mitophagy, and glycolytic enzymes—may offer multi-mechanism therapeutic strategies for liver fibrosis.

Epigenetic regulation plays a critical role in liver fibrosis as well. Downregulation of miR-4524a-5p in macrophages leads to TBP overexpression, which triggers β-TrCP-mediated ubiquitination and membrane translocation of TIM3. This process activates the PI3K/mTOR pathway, promotes M2 polarization and TGF-β release, and ultimately accelerates fibrosis in non-alcoholic fatty liver disease (177). On the other hand, the histone methyltransferase ASH1L enhances transcriptional expression of CCL2 and CSF1 through H3K4me3 modification, thereby recruiting M2-type TAMs and fostering an immunosuppressive microenvironment that promotes the progression of fibrosis-associated hepatocellular carcinoma (178). Thus, targeting the miRNA–epigenetic interplay network and histone-modifying enzymes may offer novel therapeutic strategies to suppress malignant transformation in liver fibrosis and modulate the immune microenvironment.

#### 4.2.2 Pulmonary fibrosis

During pulmonary fibrosis progression, macrophage polarization promotes disease progression through signal pathway interaction and metabolic reprogramming. Icariside II significantly alleviates bleomycin-induced pulmonary fibrosis in mice by suppressing the PI3K/Akt/β-catenin signaling axis and downregulating M2 markers such as CD206 and Arg-1, thereby reducing macrophage-mediated fibroblast activation and collagen deposition (179). Notably, a critical vicious cycle forms between macrophages and fibroblasts, macrophages induce IL-6 secretion from fibroblasts via P2rx4 signaling, which further upregulates Arg1 expression in macrophages. The ornithine generated by Arg1 metabolism is utilized by fibroblasts as a substrate for proline synthesis, ultimately exacerbating collagen production (180). Thus, targeting key signaling axes related to macrophage polarization such as PI3K/Akt/β-catenin, along with interaction pathways with fibroblasts such as P2rx4/IL-6/Arg1, may enable synergistic intervention against this disease.

The sphingolipid signaling pathway plays a pivotal role in regulating pulmonary fibrosis. Sphingosine-1-Phosphate Receptor 3 (S1pr3) is specifically upregulated in M2 macrophages and its deficiency attenuates IL-4-induced M2 polarization by blocking the PI3K/Akt/STAT3 pathway, thereby alleviating fibrotic phenotypes. S1pr3 inhibitors such as CAY10444 and TY52156 demonstrate therapeutic promise (181). Conversely, activation of G protein-coupled receptor 40 (GPR40) suppresses M2 polarization via inhibiting the PKD1/CD36/TGF-β1 axis, and its agonist SC—1,3-dihydroxy-8-methoxy-9H-xanthen-9-one—markedly mitigates lung fibrosis in mice (182). Therapeutic strategies targeting the microenvironment have also shown progress in pulmonary fibrosis treatment. Lung decellularized matrix hydrogel locally delivered to the lung downregulates the ficolin signaling pathway, suppresses M2 polarization, and reduces CD3^+^ T cell infiltration, thereby reversing bleomycin-induced pulmonary fibrosis in rats (183). Metabolically, the 11β-HSD1 inhibitor J2H-1702 upregulates heme oxygenase-1, inhibits reactive oxygen species-mediated DNA damage, and synergistically blocks endothelial–mesenchymal transition along with pro-inflammatory macrophage polarization, ultimately enhancing the anti-fibrotic efficacy of nintedanib (184). Thus, targeting immune microenvironment remodeling, such as with matrix hydrogels, in combination with oxidative stress modulation via 11β-HSD1 inhibition, offers a multi-scale therapeutic approach for pulmonary fibrosis.

#### 4.2.3 Muscle and adipose tissue fibrosis

Tissue fibrosis, characterized by the excessive deposition of extracellular matrix components, is a common pathological outcome in numerous chronic diseases. Within both muscle and adipose tissue, macrophages, as key immune cells, play a complex and central role in the initiation, perpetuation, and resolution of fibrotic processes, a function underpinned by their remarkable heterogeneity and plasticity.

In pathological muscle environments, such as Duchenne muscular dystrophy (DMD) and myocardial infarction, macrophages exhibit distinct pro-fibrotic phenotypes. Single-cell transcriptomic studies have revealed a macrophage subpopulation, abundant in dystrophic muscle, that is characterized by high expression of Galectin-3 and Secreted Phosphoprotein 1 (SPP1) and does not conform to the traditional M1/M2 classification (185). These SPP1^+^ macrophages drive fibrosis by secreting their encoded protein, osteopontin (OPN), which interacts with CD44/integrin receptors on stromal progenitor cells, such as fibro-adipogenic progenitors (FAPs), thereby promoting their differentiation into matrix-producing fibroblasts (185). Furthermore, in myocardial infarction models, a hypoxic milieu induces M2-like macrophages to express V-set and Immunoglobulin Domain Containing 4 (VSIG4), consequently facilitating the transformation of cardiac fibroblasts into myofibroblasts and leading to cardiac fibrosis (186). Notably, this SPP1-CD44/integrin axis of cellular crosstalk is not restricted to muscular tissues; it is also operational in the coronary perivascular adipose tissue (PVAT) of patients with atherosclerosis, where SPP1^+^ macrophages similarly exacerbate local fibrosis (187), revealing a common trans-tissue mechanism of macrophage-driven fibrosis. Building upon these mechanistic insights, novel targeted therapeutic strategies are emerging with significant potential. For instance, chimeric antigen receptor macrophages (CAR-Ms) targeting fibroblast activation protein (FAP) can effectively phagocytose activated cardiac fibroblasts, markedly attenuating cardiac fibrosis and improving function following myocardial ischemia-reperfusion injury (188, 189).

In the context of obesity, the aberrant expansion of white adipose tissue (WAT) is accompanied by extensive macrophage infiltration and activation, which drives chronic inflammation and fibrotic progression. During aging, the accumulation of immunoglobulin G (IgG) in adipose tissue activates the Ras signaling pathway within macrophages, inducing their secretion of TGF-β and subsequently promoting WAT fibrosis via the SMAD pathway (190). The cytokines IL-13 and IL-4, signaling through the IL4Rα receptor on macrophages, have been identified as critical mediators in inducing WAT fibrosis; this pro-fibrotic effect is dramatically diminished upon macrophage depletion (191). Single-cell analyses have further elucidated the complex intercellular communication between macrophages and fibroblasts in obese adipose tissue. A key discovery is that, beyond the previously reported lipid-associated macrophages (LAMs), a novel macrophage subpopulation expressing Macrophage-Inducible C-Type Lectin (Mincle) plays a dynamic regulatory role in fibrosis by secreting Oncostatin M (Osm), which negatively regulates collagen gene expression (192). Additionally, macrophages can directly impede preadipocyte differentiation and stimulate the expression of pro-fibrotic factors such as α-smooth muscle actin (α-SMA), prompting a myofibroblast-like phenotype, an effect that is potentiated by excess glucocorticoid exposure (193). Regarding therapeutic interventions for adipose tissue fibrosis, the phytochemical Sulforaphane has been shown to mitigate fibrosis by promoting an M2-polarized macrophage phenotype via activation of the Nrf2 pathway (194), while exercise training can attenuate diet-induced WAT fibrosis in obese mice by reducing TGF-β levels and macrophage infiltration (195). More cutting-edge approaches, such as the use of nanomaterials to scavenge cell-free nucleic acids (cfNAs) and thereby inhibit the TLR7/9-NF-κB signaling pathway, can reduce the population of pro-fibrotic Gal3^+^ macrophages (196), offering a novel strategy for combating refractory muscle fibrosis.

In conclusion, macrophages are far from being passive bystanders in fibrosis; they are active regulators. Through specific subpopulations (e.g., SPP1^+^, Gal3^+^, Mincle^+^ macrophages) and signaling molecules (e.g., OPN, TGF-β, OSM), they engage in sophisticated dialogue with stromal progenitor cells and fibroblasts, collectively determining the trajectory of the fibrotic process. Therapeutic strategies targeting these specific mechanisms and cellular interactions hold significant promise for reversing fibrosis in both muscle and adipose tissue.

### 4.3 Cancer

#### 4.3.1 TAMs and immunosuppression

TAMs shape an immunosuppressive microenvironment by polarizing towards the M2 phenotype, a process driven by metabolic reprogramming, dysregulated signaling pathways, and intercellular interactions. Lactate secreted by colorectal cancer cells induces histone H3K18 lactylation, which upregulates VSIG4 expression. This in turn activates the JAK2/STAT3 pathway, promoting FAO and thereby driving M2 polarization while enhancing PD-L1-mediated T cell exhaustion (197). Meanwhile, the SENP1/Sirt3 axis facilitates mitochondrial acetyl-CoA synthesis and cholesterol biosynthesis, inducing M2 polarization and suppressing CD8^+^ T cell function (198). In glioma, ALOX5 activates the NRF2 signaling pathway, upregulating PD-L1 expression in TAMs and promoting their M2 polarization, thereby establishing a positive feedback loop that supports immune escape (199). Collectively, these studies highlight metabolic remodeling as a central driver of TAM-mediated immunosuppression.

In triple-negative breast cancer, macrophages and cancer cells jointly activate IL1R2, which alleviates suppression of c-Fos by degrading YY1, thereby upregulating PD-L1 expression and enhancing immunosuppression (200). Within the bone metastasis microenvironment of prostate cancer, endothelial-osteoblastic transformation promotes M2 polarization of TAMs via Wnt signaling and facilitates the secretion of factors such as CXCL14 to suppress CD8^+^ T cell function (201). Notably, in glioma, the MALT1 protease enhances the immunosuppressive phenotype of TAMs through NF-κB pathway activation, while MALT1 inhibitors reverse this effect and restore antitumor immune responses (202). These findings collectively demonstrate that multiple signaling pathways form a cascade network that functions as a central regulatory hub for immune escape.

Exosome-mediated intercellular communication plays a crucial role in TAM-driven immunosuppression. In ovarian cancer, tumor cell-derived exosomes carrying CMTM4 are taken up by macrophages, promoting TGF-β1 and CXCL12 secretion via NF-κB pathway activation and enhancing ICAM1/CD206-mediated M2 polarization, thereby reducing the efficacy of PD-1 inhibitors (203). Under hypoxic conditions in glioma, tumor exosome-derived miR-25-3p drives M2 polarization of TAMs by suppressing PHLPP2 and activating the PI3K/AKT/mTOR pathway, accelerating tumor progression (204). Thus, targeting tumor exosomes and their cargo may offer a promising strategy to reverse TAM-related immunosuppression and develop novel immunotherapeutic approaches.

#### 4.3.2 Dynamic evolution of M1 and M2 macrophages in tumor progression

During tumor progression and metastasis, the dynamic equilibrium between M1 and M2 macrophages exhibits notable spatiotemporal heterogeneity. In early tumor stages, M1 macrophages exert anti-tumor effects through pro-inflammatory cytokine production, such as TNF-α and IL-1β, and antigen presentation, yet their functions are often compromised by immunosuppressive mechanisms within the TME. For instance, in lung cancer, Integrin β8 (ITGβ8) induces CCL5 secretion via the PI3K/AKT/IRF9 pathway, thereby driving M2 polarization and establishing a pro-tumorigenic feedback loop (205). Additionally, tumor-derived lactate activates cancer-associated fibroblasts, prompting IL-8 secretion that recruits M2 macrophages and further accelerates lung cancer progression (206). In contrast, M2 macrophages dominate in advanced tumor stages and promote metastasis. In bladder cancer, SPOP deficiency induces M2 polarization via CCL2, with subsequent IL-6 secretion significantly enhancing cancer stem cell properties (207). In pancreatic cancer, CCCTC-binding factor (CTCF) cooperatively regulates histone lactylation and m6A modification through lncRNA FLG-AS1, activating the IGF2BP2/CSF1 axis to drive M2 polarization (208). Semaphorin 3D (SEMA3D) increases lactate secretion via a KRAS mutation-dependent ADP-ribosylation factor 6 (ARF6) signaling pathway and induces pro-metastatic M2 polarization through the G protein-coupled receptor 132 (GPCR132) (209). Thus, targeting the CCL5/IL-8 signaling axis and related epigenetic-metabolic networks, such as histone lactylation and IGF2BP2, may disrupt M2 polarization cascades and restore anti-tumor immune balance.

Metabolic reprogramming serves as a core mechanism driving M2 polarization of TAMs. In lung cancer, Aquaporin 3 (AQP3) promotes M2 polarization and upregulates IL-6 secretion via the PPARγ/NF-κB signaling axis, thereby exacerbating glucose metabolic disorders through the IL-6R pathway (210). In the gastric cancer microenvironment, cancer-associated fibroblasts upregulate Nicotinamide N-methyltransferase (NNMT) through FTO-mediated m6A demethylation, inducing M2 polarization and suppressing CD8^+^ T cell function (211). In osteosarcoma, migrasomes carrying Milk fat globule-Epidermal growth factor-factor 8 (MFGE8) drive M2 polarization by enhancing phagocytosis, thereby establishing a pro-metastatic niche (212). During metastasis, M2 macrophages play particularly prominent roles. In a model of colorectal cancer liver metastasis, tumor-resident microbiota promotes M2 polarization and immunosuppression via lactate-induced RIG-I lactylation, which inhibits the NF-κB/NLRP3 pathway (140). In pancreatic cancer, MLKL-mediated necroptosis cooperatively facilitates metastasis through CD47 signaling and macrophage extracellular traps (213). In summary, disrupting the M2-polarizing cascades, particularly through targeting key signaling axes and metabolic-epigenetic networks, provides a spatiotemporally specific approach to reprogram the TME and enhance anti-tumor immunity.

### 4.4 Metabolic diseases

#### 4.4.1 Obesity-induced adipose tissue inflammation

In obesity, adipose tissue inflammation is characterized by macrophage infiltration and polarization imbalance, which directly contributes to metabolic abnormalities. Adipocyte-derived exosomes act as critical messengers in this process. For instance, microRNA-1224, highly expressed in adipose tissue of obese mice, inhibits M2 macrophage polarization and promotes inflammatory cytokine release by suppressing MSI2 and consequently blocking the Wnt/β-catenin pathway (214). Additionally, intrinsic stress responses in adipocytes, particularly ERS, profoundly influence macrophage behavior. The transcription factor Hoxa5 alleviates ERS by inhibiting the eIF2α/PERK signaling pathway and synergistically activates PPARγ signaling, thereby promoting M2 polarization and reducing inflammation (215). At the epigenetic level, elevated microRNA-802 in adipocytes sustains NF-κB pathway activation through TRAF3 inhibition, enhancing chemokine expression and M1 polarization, while its specific knockout ameliorates insulin resistance (216). Notably, aging-related mechanisms are also involved. The loss of miR-145-5p in exosomes from adipose progenitor cells in middle-aged individuals reduces its inhibitory effect on L-selectin and NF-κB in macrophages, exacerbating M1 polarization. Exogenous supplementation of miR-145-5p mimics improves obesity in middle-aged mice (217). These mechanisms highlight multiple molecular targets with therapeutic potential for obesity-associated inflammation.

Macrophage polarization is further regulated by metabolic reprogramming. Mitochondrial autophagy dysfunction represents a critical mechanism. In hypoxic obese adipose tissue, HIF1α-induced mitophagy receptor BNIP3 drives macrophages toward an M1 phenotype and enhances glycolysis, thereby exacerbating inflammation. Myeloid-specific deletion of BNIP3 ameliorates obesity-associated insulin resistance (218). Amino acid metabolism also contributes to this regulation. SUCLA2-mediated glutaminolysis and AMPK activity mutually constrain each other. Loss of AMPKα reduces SUCLA2 phosphorylation, promoting succinate and IL-1β production and amplifying inflammatory responses (219). Deficiency in the selenoprotein SelenoM induces mitochondrial ROS accumulation via the Hippo–YAP/TAZ axis, synergistically activating NF-κB and aggravating adipocyte inflammation along with M1 polarization (220). Fatty acid metabolism plays a significant role as well. In myeloid cells, ABCG1 modulates membrane fluidity and inflammatory intensity by regulating the distribution of saturated fatty acids between membrane phospholipids and lipid droplets. However, its absence unexpectedly alleviates adipose inflammation and insulin resistance in obese mice by suppressing lipoprotein lipase activity (221). Furthermore, low-cytotoxicity interferon tau (IFNT) regulates macrophage activation states, increases the M2 population, and improves systemic insulin sensitivity (222). Meanwhile, interferon regulatory factor IRF4 acts as a negative inflammatory regulator. Myeloid-specific deletion of IRF4 enhances M1 polarization and impairs insulin signaling (223). Collectively, these findings illuminate the complex cellular and molecular regulatory networks in adipose tissue under obesity, providing a theoretical foundation for precise intervention strategies targeting macrophage polarization.

#### 4.4.2 Diabetes and complications

Macrophage polarization imbalance plays a pivotal role in the development and progression of diabetes and its complications. The hyperglycemic microenvironment reprograms macrophage metabolism and epigenetic states, thereby modulating their functionality. For instance, in diabetic retinopathy, upregulated circular RNA circSPECC1 enhances GPX2 mRNA stability by recruiting eIF4A3, altering arachidonic acid metabolism to produce 12-HETE, which mediates macrophage-endothelial cell interactions and exacerbates microvascular dysfunction (224). In diabetic cardiomyopathy, macrophage-derived S100A9 activates the STAT3 pathway, disrupts mitochondrial quality control in cardiomyocytes, and impairs cardiac function (225). Furthermore, RasGRP4 promotes M1 macrophage polarization and Th17 cell differentiation during diabetic renal ischemia-reperfusion injury, amplifying inflammatory responses and renal fibrosis via the IL-17 signaling pathway (226). Targeting macrophage polarization has emerged as a promising therapeutic strategy for diabetic complications. Tetrahedral framework nucleic acids (Tsa) carrying SIRT1-activating RNA inhibit the NF-κB pathway through SIRT1 activation, promote M2 polarization, and improve the osteoimmune microenvironment in diabetic osteoporosis (227). Eicosapentaenoic acid upregulates HO-1 via the GPR120 receptor, inducing the Mox anti-inflammatory phenotype and mitigating hyperglycemia-induced myocardial injury (228). Collectively, these findings unveil the multidimensional regulatory network of macrophage polarization in diabetes and its complications, offering new avenues for precise immune intervention.

Modulating macrophage polarization holds significant therapeutic value in diabetic wound healing. CGRP which is downregulated in diabetic wounds promotes M2 polarization and angiogenesis by suppressing the p53 pathway. A GelMA hydrogel loaded with CGRP enables sustained peptide release and accelerates wound healing (229). A biphasic drug-delivery microneedle system enhances M2 polarization through early release of ROS-scavenging cerium oxide nanoparticles followed by sustained VEGF release to promote vascularization (230). A Janus lipase-mimetic material leveraging selenoenzyme activity clears ROS and stimulates IL-17 secretion from γδ T cells thereby shifting M1 toward M2 polarization and improving infected wound healing (231). Glycosaminoglycans derived from Andrias davidianus skin induce anti-inflammatory phenotypes marked by CD206 and Arg1 expression through modulation of macrophage glycolipid metabolism promoting tissue regeneration (232). Notably keratinocytes in diabetic wounds upregulate NLRP3 expression in macrophages via JMJD3-mediated demethylation driven by IL-1α/IL-1R signaling exacerbating inflammation which suggests JMJD3 as a potential intervention target (233). Thus, macrophage polarization in diabetes and its complications is regulated through multidimensional mechanisms and targeting polarization states along with metabolic reprogramming offers promising therapeutic strategies.

### 4.5 Neurodegenerative diseases

#### 4.5.1 Microglial polarization in AD/PD

In Alzheimer’s disease (AD) and Parkinson’s disease (PD), disrupted microglial polarization is a central mechanism driving neuroinflammation and neurodegeneration. In AD research, shifting microglia from the pro-inflammatory M1 phenotype to the anti-inflammatory M2 type can effectively delay disease progression. For example, berberine binds and activates TYROBP, stabilizing its oligomeric form, enhancing microglial phagocytosis of Aβ, and promoting M2 polarization, thereby improving cognitive function (12). The circular RNA circAPP is highly expressed in the hippocampus of AD mice and exacerbates M1 polarization by competitively binding miR-1906 and upregulating CLIC1. Inhibiting circAPP or overexpressing miR-1906 reverses polarization imbalance and alleviates pathology (234). Neuronal damage can also indirectly regulate microglial phenotype. Schisandrin B inhibits GSK3β and activates the Nrf2/GPX4 pathway, reducing neuronal ferroptosis and consequently blocking TNF-α-mediated M1 polarization (235). Environmental factors such as PM_0.1_ exposure downregulate circ_0061183, which relieves inhibition of miR-98-5p and impairs IL-10-driven M2 polarization. This mechanism has been validated in the blood of AD patients (236). Non-invasive physical interventions like rotating magnetic fields promote the transition from M1 to M2 by suppressing the NF-κB/MAPK pathway and enhancing Aβ clearance (237).

In PD, microglial polarization is closely linked to metabolic reprogramming. Iron accumulation and inflammasome activation promote a glycolytic shift, driving M1 polarization, while M2-type cells are more susceptible to ferroptosis (238). Dectin-1 and TLR4 collaboratively sustain the M1 phenotype through the Syk/NF-κB axis, and their inhibitor xylan alleviates dopaminergic neuronal damage (239). Epigenetic mechanisms such as lncRNA HOXA-AS2 mediate methylation of the PGC-1α promoter via the PRC2 complex, suppressing its expression and facilitating M1 polarization (240). Pathological α-synuclein fibrils combined with TNF-α and PGE2 induce a toxic microglial phenotype characterized by enhanced glutamate release and Slc7a11 upregulation, exacerbating neuronal injury (241). Apilarnil reestablishes polarization balance by synergistically downregulating M1 markers (miR-155/CD36/CXCL10) and upregulating M2 markers (miR-124/CD206/arginase 1) (242). Eprosartan and xanthenone promote STAT6 phosphorylation through the MKP-1/miR-155/SOCS1 axis and PP2A signaling, thereby driving M2 polarization (243). Furthermore, loss of c-Cbl exacerbates M1 polarization and accelerates neurodegeneration via the PI3K/Akt pathway, while its functional restoration reverses this process (244). In Parkinson’s disease models, dopamine has been found to upregulate iron transporters on the cell membrane (such as TFR1, ferroportin), thereby promoting cellular iron uptake. This discovery reveals a previously unknown direct link between neurotransmitters and iron metabolism, suggesting that in brain regions rich in dopaminergic neurons, iron metabolism in macrophages/microglia may be uniquely regulated by the local neurochemical environment (245). Therefore, targeting core mechanisms including microglial metabolic reprogramming, key receptor signaling, epigenetic regulation, and toxic phenotype induction to rebalance polarization represents a critical strategy for mitigating neuroinflammation and neurodegeneration in PD.

Collectively, a multidimensional approachensionalnTA a natural compounds, RNA modulation, neuronal protection, physical intervention, and metabolic reprogrammingncan precisely modulate microglial polarization. Clearing pathological protein aggregates, targeting metabolic processes, and regulating non-coding RNAs thereby emerge as key strategies for neuroimmunological intervention in AD and PD.

#### 4.5.2 Neuroinflammation in MS and stroke

In both multiple sclerosis (MS) and ischemic stroke, neuroinflammation is driven by an imbalance in the polarization of microglia and macrophages, yet the pathological context and regulatory strategies differ substantially. In MS, a chronic autoimmune disorder, macrophages exhibit intrinsic metabolic defects, particularly impaired mitochondrial energy metabolism, which sustains a pro-inflammatory state even under homeostatic or reparative signals. This dysfunction is manifested through increased secretion of cytokines and chemokines, reduced capacity for myelin clearance, and the release of supernatants that promote astrocyte differentiation rather than oligodendrogenesis. These alterations are closely associated with the enrichment of CD16^+^ pro-inflammatory subsets within MS lesions (246). Consequently, therapeutic strategies focus on rectifying metabolic abnormalities. For instance, paeoniflorin directly binds to the glycolytic enzyme ENO1, inhibiting its activity and expression, thereby disrupting glucose metabolism and suppressing M1 polarization in microglia and macrophages, which alleviates central inflammation in experimental autoimmune encephalomyelitis (247). Thus, targeting intrinsic metabolic dysfunctions in macrophages to restore their responsiveness to reparative signals represents a core strategy for intervening in MS pathogenesis.

In contrast, neuroinflammation following ischemic stroke manifests acutely, with regulatory strategies focusing on shifting microglia/macrophages from the M1 to the M2 phenotype. For instance, early administration of the Annexin A1 bioactive peptide Ac2–26 during reperfusion activates the FPR2/ALX receptor and triggers the AMPK/mTOR signaling axis. This promotes M2 polarization in the ischemic penumbra, attenuating blood-brain barrier disruption and neuronal apoptosis, a mechanism correlated with favorable clinical outcomes (248). FGF21 suppresses NF-κB while upregulating PPARγ, modulating polarization dynamics, curbing M1 polarization and peripheral immune cell infiltration, thereby improving neurological function (249). Metabolic reprogramming represents another key intervention target. Genetic deletion of Sult2b1 impairs cholesterol sulfate synthesis and exacerbates pro-inflammatory polarization, whereas exogenous cholesterol sulfate reverses this effect by modulating NADPH/ROS levels and the AMPK/CREB pathway (250). Additionally, sulfated chitosan (SCS) induces neutrophil apoptosis and promotes M2 macrophage polarization via the IL-10/STAT3 pathway, shortening the acute inflammatory phase and facilitating tissue repair (251) Thus, post-stroke remodeling of microglial/macrophage polarization—through activating specific receptor pathways such as FPR2/ALX and PPARγ, regulating metabolic programs like cholesterol sulfate synthesis, and modulating immune cell crosstalk—constitutes a critical strategy for mitigating secondary injury and promoting neural recovery.

Consequently, neuroinflammation in multiple sclerosis originates from a chronic pro-inflammatory state caused by intrinsic metabolic defects in macrophages, while post-stroke inflammation emphasizes the dynamic transition of polarization phenotypes during the acute phase. Together, these findings demonstrate that targeting cellular metabolic reprogramming, such as glycolysis and cholesterol metabolism, along with key receptor signaling pathways, represents an effective strategy for modulating neuroimmune balance.

### 4.6 Infection

During infection, the state of macrophages is precisely regulated by a variety of mechanisms, which collectively determine the outcome of the host immune response. Specifically, pathogens such as cytomegalovirus can hijack host signaling pathways (e.g., Wnt/ZEB1) to reprogram macrophage identity, inducing stem cell-like characteristics, enhanced migration and invasiveness, while impairing their inherent tissue immune functions (252). Furthermore, pathogens can manipulate macrophage metabolism to promote their own survival; for instance, *Salmonella* infection preferentially utilizes M2b-polarized macrophages and activates PPARα to promote fatty acid oxidation, thereby establishing intracellular infection (253). In fact, the metabolic reprogramming of macrophages is closely linked to their antimicrobial functions and engages in a continuous “evolutionary tug-of-war” with bacterial metabolic adaptations (254). Moreover, tissue-resident macrophages exhibit significant metabolic heterogeneity both at homeostasis and during infection. For example, during helminth infection, newly recruited monocyte-derived macrophages undergo metabolic reprogramming and alternative activation, while TIM4+ resident macrophages remain immunologically and metabolically hyporesponsive (255). On the other hand, some bacteria have evolved sophisticated immune evasion strategies; for instance, a prophage competition element in *Salmonella* cleaves a subset of tRNAs to inhibit the lytic cycle, thereby reducing the release of immunogenic components and altering the macrophage response to infection (256). Similarly, the SaeRS two-component system of *Staphylococcus aureus* promotes bacterial clumping in the early stage of infection, which masks antigens and subsequently inhibits NF-κB pathway activation in macrophages, ultimately impairing their phagocytic, bactericidal, and cytokine-producing capacities (257).

In the context of viral infections, influenza virus can infect alveolar macrophages, leading to persistent inflammation, reduced phagocytic function, mitochondrial damage, metabolic alterations, and potentially even the “macrophage disappearance reaction” (258). The protein CDC42-165aa, encoded by circular RNA circCDC42, inhibits CDC42 GTPase activity, thereby activating the Pyrin inflammasome and leading to pyroptosis of alveolar macrophages in *Klebsiella pneumoniae* infection, which exacerbates lung injury (259). Furthermore, certain infections, such as *Acinetobacter baumannii* sepsis, can drive alveolar and interstitial macrophages to polarize into an M1 phenotype and trigger a lethal cytokine storm via the TLR2/MyD88/NF-κB pathway (260). Finally, macrophage subsets in different tissue locations exhibit functional specificity in their responses to infection. For example, in the bladder, MacM (muscularis) and MacL (lamina propria) macrophages display distinct fates and functions during urinary tract infection: MacM exhibits stronger phagocytic ability and anti-inflammatory polarization, whereas MacL undergoes rapid death (261). Based on these mechanisms, developing strategies that target and modulate macrophage states holds therapeutic potential. For instance, pH-responsive nanoparticles can effectively clear intracellular bacteria by disrupting bacterial cell walls and promoting macrophage polarization towards the M1 phenotype (262). In conclusion, these studies profoundly reveal that macrophages are not passive responders but rather the core battlefield in the host-pathogen interplay during infection. A deeper understanding of the complex network regulating their state will lay a solid theoretical foundation for developing novel immunotherapeutic strategies against infectious diseases.

## 5 Therapeutic targeting of macrophage polarization

The precise modulation of macrophage polarization states has emerged as a novel therapeutic strategy for various diseases, with core approaches encompassing pharmacological intervention, immunotherapy, gene editing, and cell-based therapies. Pharmacological agents target key signaling pathways such as JAK/STAT and PPARγ or regulate metabolic processes including glycolysis and FAO to remodel the immune microenvironment. CAR-M therapy combined with engineered exosome technology enhances targeting ability and immune synergy, significantly improving antitumor efficacy. CRISPR-Cas9 and miRNA editing enable precise regulation of polarization-related gene networks. However, these strategies face considerable challenges in clinical translation, such as the highly context-dependent effects of polarization (exemplified by the opposing roles of M2 macrophages in tissue repair versus tumor progression), off-target risks of delivery systems, and dynamic heterogeneity of biomarkers. The following sections will systematically examine the mechanistic advances and translational bottlenecks of these therapies, while also outlining future directions for spatiotemporally specific interventions.

### 5.1 Pharmacological approaches

**Figure 5:**
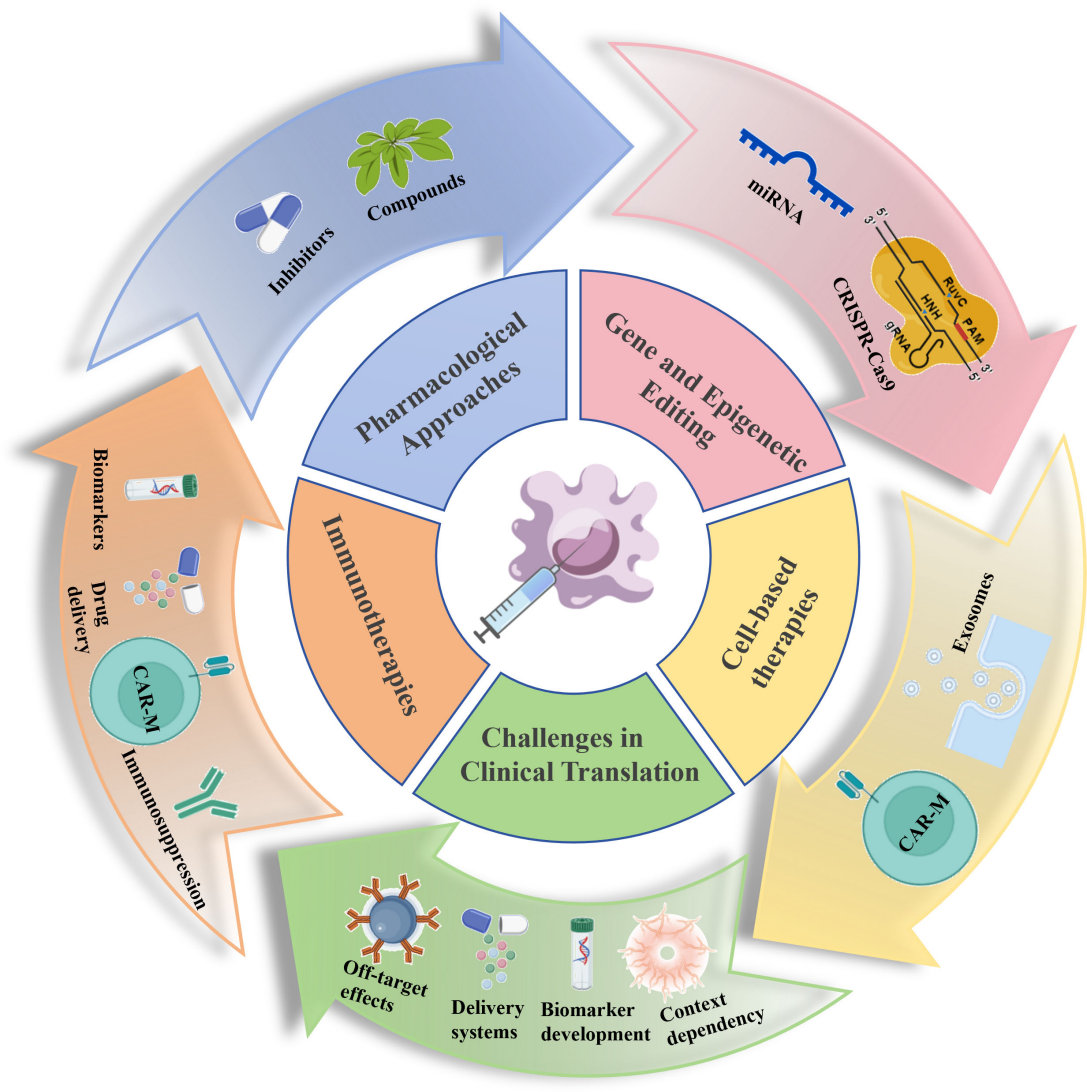
Precision modulation of macrophage polarization: emerging therapeutic strategies and translational challenges. This figure systematically summarizes four major therapeutic strategies targeting macrophage polarization: (1) pharmacological interventions, such as inhibitors, agonists, or other compounds targeting signaling pathways like JAK/STAT/PPARγ and metabolic reprogramming; (2) immunotherapy, including CAR-M combined with immunosuppressants and enhanced tumor targeting and immune activation through drug delivery systems; (3) genetic and epigenetic editing, utilizing technologies like CRISPR-Cas9 or miRNA to precisely regulate polarization-related genes; (4) cell therapy, such as CAR-M combined with engineered exosomes. These strategies aim to remodel the immune microenvironment and enhance therapeutic efficacy. However, their clinical translation faces numerous challenges, including the highly context-dependent nature of polarization effects (e.g., the dual functionality of M2 macrophages), off-target risks of delivery systems, and heterogeneity of biomarkers. Picture created using BioRender and BioGDP.

Pharmacological interventions in macrophage polarization employ diverse targeting strategies to remodel the immune microenvironment by modulating key signaling pathways and metabolic reprogramming (**Figure 5**). The ALOX5 inhibitor zileuton suppresses M2 polarization of TAMs via blocking the JAK/STAT pathway, thereby attenuating pancreatic cancer invasion and metastasis (263). Cinobufagin inhibits M2 macrophage polarization by downregulating the PPARγ/MARCO axis, consequently suppressing lung cancer cell migration (264). Inhibition of the RUNX1/SLAMF3 axis restores phagocytic dysfunction in TAMs during colorectal cancer liver metastasis, increases the proportion of C1QC^+^TAMs, and alleviates T cell exhaustion (265). Shengmai San Formula (SMS) modulates gut microbiota to elevate conjugated bile acids such as TCA, which stimulates Slit3 release from M2 macrophages in adipose tissue, activates the sympathetic nerve-norepinephrine axis, and promotes white adipose browning along with energy expenditure (266). PPARγ agonists play central regulatory roles across multiple disease models. Bexarotene activates the PPARγ/HO-1 pathway, ameliorating cigarette smoke-induced M1 polarization in alveolar macrophages and reducing lung inflammation (267). 15(S)-HETE drives peritoneal macrophages toward an anti-tumor phenotype via the PPARγ/C/EBPβ axis (268), while genipin inhibits CCR2 signaling through PPARγ-mediated p65 degradation, reducing macrophage infiltration and preventing hepatocellular carcinoma recurrence (269). These findings collectively demonstrate that targeting signaling pathways such as JAK/STAT and PPARγ represents a promising strategy for modulating macrophage polarization and related diseases.

Metabolic reprogramming represents another critical intervention strategy. Metformin-derived carbon dots facilitate macrophage transition to the M2 phenotype by scavenging ROS and suppressing the NLRP3 inflammasome, while simultaneously blocking the IL-6/gp130 pathway to restore fibroblast homeostasis (270). SGLT2 inhibitors such as dapagliflozin and canagliflozin target the glycolytic enzyme PFKFB3, promoting a shift from M1 to M2 polarization through metabolic reprogramming and thereby ameliorating steatosis and fibrosis in non-alcoholic fatty liver disease (176). Xianglian pill elevates itaconate levels and inhibits the TET2/STAT1-NFκB pathway to suppress M1 polarization, alleviating ulcerative colitis (151). Pentose phosphate pathway inhibitors enhance macrophage phagocytosis of lymphoma cells by suppressing the UDPG/STAT1/IRG1/itaconate axis (79). Astragaloside IV binds to the mitochondrial citrate carrier Slc25a1, restoring the TCA cycle and promoting M2 polarization, which attenuates neuroinflammation after stroke (271). The traditional Chinese medicine formula KAJF modulates gut microbiota and the metabolite butyrate to inhibit M2 polarization and enhance CD8^+^ T cell infiltration, suppressing liver metastasis formation (272). Additionally, natural compounds like shikonin promote a metabolic shift from glycolysis to OXPHOS via activation of the MCU/Ca²^+^ signaling pathway, inducing M2 polarization and ameliorating acute lung injury (273). Fisetin mitigates microglial M1 polarization after spinal cord injury by inhibiting the JAK2/STAT3 pathway (274). These studies highlight the therapeutic potential of targeting metabolic enzymes, metabolites, and inflammatory signaling hubs to reprogram macrophage metabolism and polarization phenotypes.

In summary, pharmacological strategies targeting key signaling pathways and metabolic reprogramming offer novel therapeutic avenues for tumors, metabolic diseases, and inflammatory disorders by modulating macrophage phenotypes.

### 5.2 Immunotherapies

In the field of immunotherapy, precise regulation of macrophage polarization has emerged as a transformative strategy. CAR-M therapy, a representative advancement, demonstrates remarkable potential (**Figure 5**). For instance, HER2-targeted CAR-M (CT-0508) exhibited favorable safety and TME remodeling capabilities in a first-in-human clinical trial. Among 14 patients with HER2-overexpressing solid tumors, no dose-limiting toxicities or severe cytokine release syndrome were observed. At eight weeks post-treatment, 44% of HER2 3+ patients achieved stable disease, accompanied by increased CD8^+^ T cell infiltration in tumors, suggesting adaptive immune activation via antigen presentation (275). Further studies indicate that inhibiting the proprotein convertase Furin sustains the pro-inflammatory phenotype of CAR-M, enhances its phagocytic capacity against breast cancer cells and patient-derived organoids, and ameliorates the immunosuppressive microenvironment through secretion of T-cell-activating factors (276). Combination strategies further optimize immune responses via multi-target interventions. The microtubule inhibitor vinblastine drives TAMs toward the M1 phenotype by activating NF-κB and Cyba-dependent ROS pathways, while promoting TFEB nuclear translocation to enhance lysosomal function. Combined with PD-1 inhibitors, it significantly augments anti-tumor efficacy (277). In terms of epigenetic regulation, inhibiting peptidylarginine deiminase 4 (PAD4) blocks arginine citrullination of STAT1, thereby relieving PIAS1-mediated suppression of MHC-II expression. This enhances antigen presentation by TAMs and promotes T cell activation (278). Targeting PGAM5 in tumor cells reduces mitochondrial DNA stress and suppresses M2 polarization via the TLR9/NF-κB/CCL2 axis, significantly improving the efficacy of immunotherapy in liver cancer (279). In summary, the clinical potential of CAR-M therapy is becoming evident, while targeting key nodes in macrophage polarization—such as Furin, PAD4, and PGAM5—and their combination with existing therapies like PD-1 inhibitors are opening new avenues for enhancing anti-tumor immune responses.

Novel drug delivery systems have significantly improved the precision of macrophage polarization regulation (**Figure 5**). Modular DNA nanodevice agonists (DNDA) achieve selective uptake by macrophages through precise self-assembly, releasing cGAS-recognizing fragments intracellularly to activate the cGAS–STING pathway, thereby synergizing with immune checkpoint blockade to exert antitumor effects (280). Mannose-modified engineered mitochondria (mPEI/M1mt) target M2 macrophages, enhance glycolysis and ROS production, activate the NF-κB/MAPK pathway, reprogram them to an M1 phenotype, and improve the efficacy of PD-L1 blockade (281). Thermosensitive R848-loaded lipid nanoparticles combined with photothermal therapy induce immunogenic cell death in bladder cancer cells, promote TAM repolarization to M1, and synergize with PD-1 inhibitors to remodel the immune microenvironment (282). Among localized delivery strategies, simvastatin-loaded PLGA nanoparticles precisely target adipose tissue, regulate macrophage polarization, induce adipose browning, and effectively alleviate obesity-related inflammation (283). Drug-loaded liposomes achieve brain targeting via nasal delivery; curcumin nanoparticles combined with cardiolipin clear Aβ deposits and inhibit the TLR4/NF-κB pathway, promoting microglial polarization toward the M2 phenotype, thereby mitigating neuroinflammation and cognitive impairment in Alzheimer’s disease models (284). Furthermore, emodin nanocapsules targeting macrophage mannose receptors regulate lipid metabolic reprogramming to influence polarization (285), while biomimetic nanoparticles delivering SIRT1 inhibitors suppress M2 polarization via the PI3K–Akt/NF-κB pathway, preventing hepatitis B-related hepatocellular carcinoma (286). These intelligent delivery systems enhance targeting and spatiotemporal controllability, providing more precise tools for macrophage polarization intervention and improving synergistic effects in combined immunotherapies.

Biomarker-guided personalized therapy has achieved significant progress in this field (**Figure 5**). For instance, SPI1^+^CD68^+^ TAMs are established as a prognostic marker in gastric cancer. They promote angiogenesis and M2 polarization through transcriptional regulation of VEGFA. Thus, their infiltration level predicts responses to anti-angiogenesis therapy combined with immunotherapy (287). WWOX deficiency competitively binds to the NME2–KAT1 complex, enhancing oleic acid synthesis and inducing an immunosuppressive macrophage phenotype. Inhibiting SCD5 reverses resistance to immune checkpoint inhibitors in liver cancer (288). Plasma S100A1 serves as a predictive biomarker for immunotherapy response in lung cancer. Its high expression drives M2 polarization of TAMs via the USP7/p65/GM-CSF axis. Targeting this pathway promotes M1-like repolarization (289). Blocking CPA4 expression disrupts the positive feedback loop between tumor cells and M2 macrophages in thyroid carcinoma (290). Inhibiting Parkin enhances MHC-I antigen presentation and reverses T cell exhaustion. Combining Parkin depletion with immune checkpoint blockade shows potential in predicting outcomes in solid tumor patients (90). In summary, identifying key biomarkers and their regulatory mechanisms not only aids in predicting therapeutic response and patient stratification but also offers precise targets for reversing immunosuppressive polarization and overcoming drug resistance.

### 5.3 Gene and epigenetic editing

Emerging strategies in the genetic and epigenetic regulation of macrophage polarization, notably the CRISPR-Cas9 system and miRNA-based therapies, offer innovative approaches for precise immunophenotype modulation (**Figure 5**). CRISPR-Cas9 enables direct reprogramming of macrophage function through targeted gene editing. For instance, knockout of the transcriptional co-regulator CITED1 significantly suppresses pro-inflammatory gene expression such as Ccl2 and Isg15 upon IFNγ stimulation, thereby alleviating persistent inflammation (291). Conversely, a V203A point mutation disrupting TRAF1/cIAP2 interaction blocks TAK1 phosphorylation downstream of TLR4, inhibiting NF-κB/MAPK pathway activation and reducing joint inflammation in rheumatoid arthritis models (292). Advances in delivery systems further broaden its therapeutic potential. pH-responsive bacterial protoplast nanovesicles delivering CRISPR components targeting PIK3CG successfully reprogram TAMs toward an M1 anti-tumor phenotype (293), while inhalable core–shell lipid nanoparticles encapsulating HK2-targeting CRISPR/Cas9 suppress glycolytic metabolism and mitigate hyperinflammation in acute lung injury (294). Thus, CRISPR-Cas9, with its precise editing capability and continuously optimized delivery technologies, is emerging as a powerful tool for modulating macrophage polarization and treating related diseases.

MicroRNA therapeutics precisely modulate polarization balance by regulating key nodes in signaling networks. For instance, miR-369-3p reverses macrophage inflammation in diabetic atherosclerosis by targeting GPR91, thereby inhibiting the succinate/TLR9/NF-κB axis and suppressing NLRP3 inflammasome activation (295). Innovations in delivery strategies have significantly enhanced targeting efficacy. Mannose-modified exosomes deliver miR-23b-3p to alveolar macrophages, mitigating septic lung injury through suppression of the Lpar1/NF-κB pathway (296). Platelet membrane-coated nanovesicles loaded with miR-181a-5p improve cardiac targeting and promote M2 polarization to enhance post-infarction repair (297). Naturally derived miRNAs also demonstrate multi-target regulatory advantages. Tea-derived vesicles containing osa-miR166d-5p and gma-miR396a-3p promote M2 polarization and alleviate colitis by suppressing AKT1/IKBKB to attenuate NF-κB signaling (298). The circular RNA ciR-ElS sequesters miR-548m to relieve its inhibition of IGF1, driving M2 transformation and ameliorating hepatitis and lipid accumulation (299). Adipose stem cell exosomal miR-1246 promotes M2 polarization by inhibiting TRAF6, while concurrently targeting FTO and RUNX1T1 to reduce lipogenesis and induce white adipose browning, thereby synergistically ameliorating obesity (300). Thus, miRNA therapeutics, leveraging their network regulatory properties, advances in delivery technology, and multi-target synergistic effects of natural miRNAs, offer promising strategies for reversing macrophage dysregulation in diseases.

Consequently, the synergistic development of gene-editing technologies and miRNA therapeutics enables precise modulation of polarization-associated gene networks and immunometabolic crosstalk, thereby providing transformative therapeutic strategies for reprogramming inflammatory diseases and the tumor immune microenvironment.

### 5.4 Cell-based therapies

In the field of cell therapy, engineered exosomes and CAR-M technology have emerged as innovative strategies for regulating macrophage polarization (**Figure 5**). Engineered exosomes derived from M1 macrophages, displaying IL4R-targeting peptides and loaded with NF-κB p50 siRNA and miR-511-3p, specifically bind to the IL-4 receptor on TAMs, promoting their reprogramming toward an M1 phenotype, enhancing anti-tumor immunity, and suppressing tumor growth (301). Similarly, bone marrow mesenchymal stem cell exosomes co-loaded with Galectin-9 siRNA and oxaliplatin reverse the immunosuppressive phenotype of TAMs by blocking the Galectin-9/Dectin-1 axis and induce immunogenic cell death in tumor cells, synergistically improving immunotherapy efficacy in pancreatic cancer (302). In metabolic diseases, lean adipose tissue macrophage-derived exosomes (ExosLean) deliver miR-222-3p to target the inflammatory factor Bcl2l11/Bim, promoting macrophage polarization toward a reparative M2 phenotype in diabetic wounds and accelerating tissue healing (303). Netrin1-overexpressing adipose stem cell exosomes (N-Exos) promote angiogenesis via activation of the PI3K/AKT/eNOS and MEK/ERK pathways and induce M1-to-M2 transition, effectively alleviating diabetic limb ischemia (304). Notably, exosomes secreted by TAMs in the TME highly express PD-L1 and inhibit CD8^+^ T cell function through a Rab27a-dependent mechanism, while knockdown of Rab27a in macrophages significantly enhances the efficacy of anti-PD-1 therapy (305). Organelle-specific engineered vesicles demonstrate differential regulatory capacities. Endoplasmic reticulum-derived vesicles from M1 macrophages (erMEVs) induce M2-to-M1 conversion more effectively than plasma membrane-derived vesicles (pmMEVs), with differences in membrane protein composition considered key to polarization regulation; erMEVs exhibit stronger tumor-killing activity in *in vitro* co-culture models (306). Regarding chronic inflammation regulation, M2 macrophage exosomes encapsulated in degradable PEG hydrogels (Exogels) enable sustained release, promoting local macrophage transition from M1 to M2 phenotype and improving tissue repair (307). Gingival mesenchymal stem cell exosomes (GMSC-Exo) reprogram macrophage metabolism from glycolysis to OXPHOS by suppressing HIF-1α signaling, thereby promoting M2 polarization in a periodontitis model (308). Furthermore, the lncRNA HOTTIP carried by M1 exosomes competitively binds to miR-19a-3p/miR-19b-3p, activating the TLR5/NF-κB pathway, which not only suppresses head and neck cancer progression but also polarizes circulating monocytes into an anti-tumor M1 phenotype (309). Together, these studies demonstrate that engineered exosomes and specific vesicle delivery systems can precisely modulate macrophage polarization, showing broad potential in cancer immunotherapy, tissue regeneration, and anti-inflammatory applications.

CAR-M therapy demonstrates significant therapeutic value in both tumor and fibrotic disease models (**Figure 5**). CAR-M cells targeting fibroblast activation protein specifically phagocytose activated fibroblasts in myocardial ischemia-reperfusion injury, reducing fibrosis and improving cardiac function (188). For large-scale production, human iPSC-derived anti-CD19 CAR-M cells expanded in bioreactors exhibit specific phagocytic capacity against CD19^+^ leukemia cells, and single-cell sequencing confirms their ability to recruit adaptive immune cells through chemokine secretion (310). To address challenges in solid tumor treatment, bispecific CAR-M cells targeting HER2 and CD47 enhance phagocytosis of ovarian cancer cells while activating CD8^+^ T cells and remodeling the TME (81). Mechanistically, CAR-M cells carrying the FcRγ signaling domain synergize with CAR-T cells, as inflammatory cytokines secreted by CAR-T upregulate CD86/CD80 expression on CAR-M surfaces, promoting M1 polarization, whereas upregulated costimulatory molecules on CAR-M further activate CAR-T, forming a positive feedback loop that enhances antitumor activity (311). In summary, CAR-M technology shows broad prospects in targeted clearance of pathological cells, scalable manufacturing, solid tumor therapy, and immune activation, offering novel strategies for treating cancer and fibrotic diseases.

### 5.5 Challenges in clinical translation

The clinical translation of macrophage polarization therapies faces several critical challenges that require urgent resolution (**Figure 5**). Primarily, off-target effects compromise treatment safety. For instance, CD47 blockade combined with radiotherapy may activate macrophage-mediated abscopal effects and suppress metastatic lesions, yet it risks inducing systemic immune activation and uncontrolled inflammation (312). Similarly, macrophages loaded with transition metal catalyst nanozymes enable prodrug conversion at tumor sites, but their phenotypic stability during migration remains unverified (313). Furthermore, optimizing delivery systems is essential to enhance targeting precision and safety. A ROS-responsive simvastatin nanopropdrug utilizing thioketal linkers enables lesion-specific drug release, and its combination with a fibronectin-targeted co-delivery system significantly reduces off-target leakage while enhancing anti-inflammatory efficacy in atherosclerotic plaques (314). For hard-to-transfect M2 macrophages, nucleic acid delivery systems leveraging mTORC1 inhibition—such as chloroquine-loaded disulfide-bridged organosilica nanoparticles—promote M1 polarization, enhance gene delivery efficiency, and offer new strategies for anti-infection therapy (315). Self-reinforcing MOF hydrogels (KZIF@HA) injected intra-articularly enable sustained release of Kartogenin to promote cartilage regeneration and release Zn²^+^ to induce M2 polarization for microenvironment remodeling, though the toxicity of long-term metal ion accumulation requires further evaluation (316). Conversely, while matrix metalloproteinase-responsive macrophage membrane-coated nanoparticles (2-APB@DGP-MM) achieve joint-targeted delivery in rheumatoid arthritis, the shedding of membrane proteins in circulation may compromise targeting accuracy (152). Therefore, systemic optimization must comprehensively address off-target risks, carrier stability, and delivery precision.

The development of biomarkers faces considerable challenges. Spatial transcriptomic analyses reveal that DTX3L, an epithelial marker, and BST2, a stromal marker, are co-upregulated in esophageal squamous cell carcinoma, promoting M2 polarization and driving tumorigenesis, however their universality across cancer types remains to be validated (317). The ADVISE trial attempted to establish a biomarker-guided allocation algorithm for combination immunotherapy, although it achieved treatment matching within 12 days for 87% of patients, it completely failed to predict outcomes in PD-1 resistant populations, with a treatment response rate of zero, highlighting the challenges posed by the dynamic TME for precise modeling (318). Thus, overcoming cancer-type specificity limitations of biomarkers and deciphering spatiotemporal heterogeneity within the microenvironment are crucial for enhancing their predictive power.

Moreover, macrophage polarization therapy exhibits profound context-dependent effects, making its outcomes in humans challenging to predict and control precisely. Macrophage phenotypes are tightly regulated by the local microenvironment, and the same intervention may yield opposing effects in different disease contexts or tissues. For instance, M2 polarization promotes repair in tissue regeneration (319) yet exerts immunosuppressive functions in tumors. In colon cancer, PTX3 induces M2 polarization via the CREB1 pathway, and its high expression correlates with poor prognosis (320). This paradoxical role demands therapeutic strategies to be precisely tailored to specific pathological contexts. Additionally, interventions targeting key signaling molecules often lead to unintended consequences due to variations in subcellular localization or cellular states. For example, inhibiting Ras activity under specific conditions, such as uniform membrane distribution of RasGAP or rear-targeting, can enhance posterior myosin contractility, thereby accelerating cell polarization and migration (321). Yet this effect is highly spatially constrained and results in complex, unpredictable chemotactic behavior. Similarly, targeting the TGF-β pathway remains challenging owing to its strong contextual dependency. Although selective blockade of integrin αvβ8-mediated TGF-β activation has been shown to reverse tumor immunosuppression and enhance immune cell infiltration by modulating macrophage polarization, broader applications require caution (322). Notably, emerging strategies are exploring active manipulation of the microenvironment to steer polarization favorably. Macrophage-hitchhiking delivery of β-elemene-loaded GeS nanosheets effectively accumulates in tumors and remodels the immunosuppressive microenvironment under ultrasound exposure, promoting M1-like polarization (323). Likewise, radiotherapy combined with CD47 blockade elicits robust abscopal antitumor effects mediated by macrophages (312). Furthermore, mild hyperthermia dynamically regulates the iNOS/Arg1 balance in macrophages, inducing an early M1 phenotype to enhance antibacterial responses and later promoting a shift toward M2 to facilitate angiogenesis and bone repair (324), demonstrating the therapeutic potential of spatiotemporal regulation of polarization. Nevertheless, a deep mechanistic understanding of the molecular networks governing macrophage fate decisions within specific pathophysiological microenvironments remains essential for safe and effective clinical translation. This high contextual sensitivity necessitates that future therapeutic designs incorporate nuanced situational awareness and individualized approaches.

Therefore, successful clinical translation of macrophage polarization therapies requires the development of spatiotemporally controllable delivery systems, the establishment of dynamic biomarker profiles, and a deeper investigation into disease-specific polarization mechanisms, with the ultimate goal of achieving precise immune remodeling.

## 6 Emerging technologies and future directions

**Figure 6:**
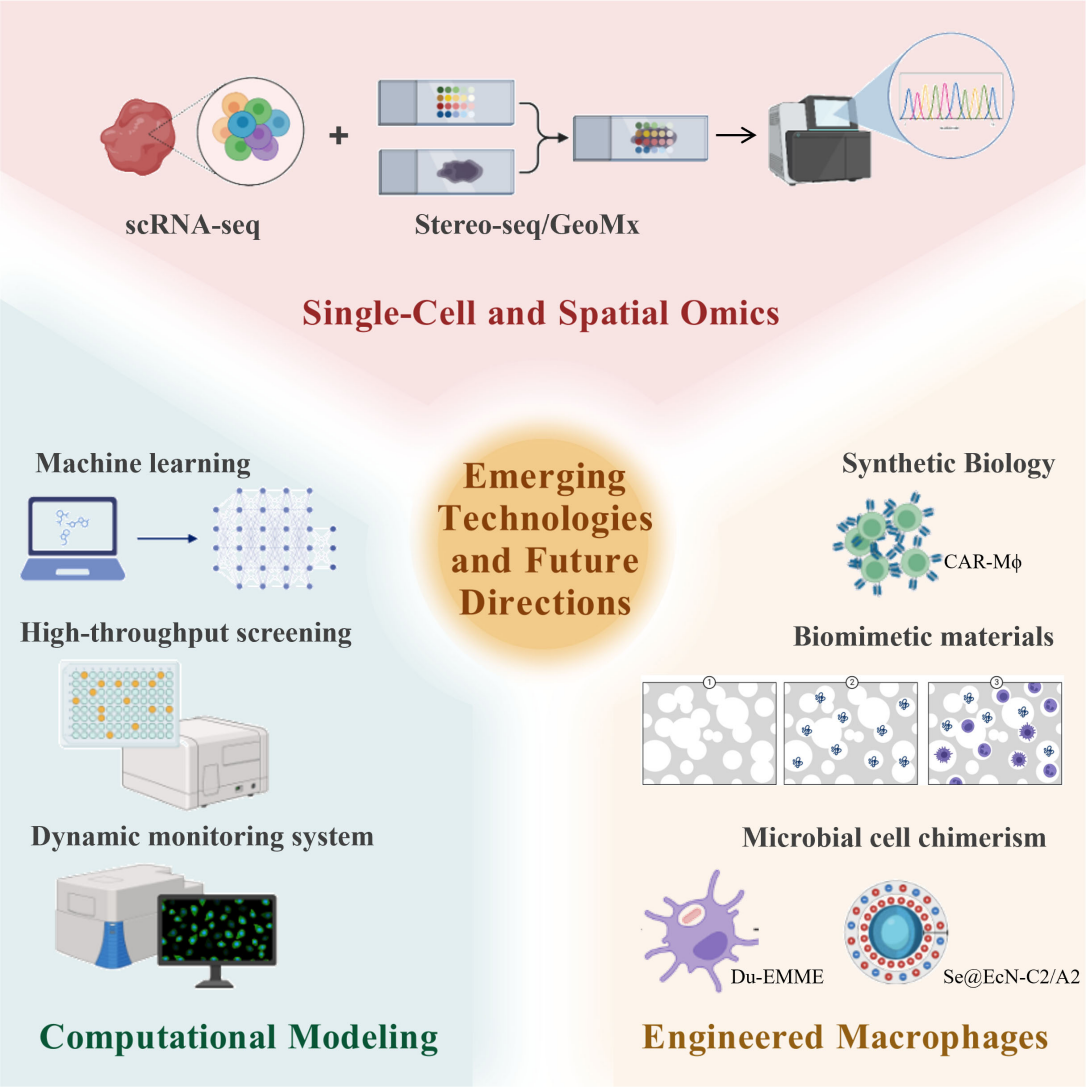
Technological integration and future directions in macrophage research. This framework comprises three interconnected modules: (1) Single-Cell and Spatial Omics: utilizing technologies such as scRNA-seq, Stereo-seq, and GeoMx to decipher macrophage heterogeneity and polarization states, with spatial transcriptomic data being integrated into computational modeling platforms; (2) Computational Modeling: combining machine learning, high-throughput screening, and dynamic monitoring systems to advance intelligent design processes; (3) Engineered Macrophages: achieving precise immune programming through synthetic biology strategies (e.g., CAR-M), biomimetic materials, and microorganism-cell hybrid systems (e.g., the dual-engine Du-EMME platform and engineered bacterial systems like Se@EcN-C2/A2). These three modules operate synergistically via a closed-loop data flow, collectively accelerating the clinical translation of macrophage-targeted therapies. Picture created using BioRender and BioGDP.

Macrophage research is undergoing a paradigm shift driven by multidisciplinary integration, supported by three cutting-edge technologies—single-cell and spatial omics, computational modeling, and engineered reprogramming. These approaches systematically overcome the limitations of traditional research through a closed-loop framework encompassing data decoding, intelligent prediction, and precise intervention. Specifically, single-cell and spatial omics resolve macrophage heterogeneity across temporal and spatial dimensions with nanoscale resolution. Computational modeling integrates multi-scale data to construct dynamic networks capable of predicting polarization states. Engineering technologies enable on-demand reprogramming of macrophage function. Together, these strategies synergize to advance the field from mechanistic investigation toward personalized therapeutics, opening new avenues for treating diseases such as cancer, fibrosis, and chronic inflammation (**Figure 6**).

### 6.1 Single-cell and spatial omics

Recent advances in single-cell and spatial omics technologies have provided powerful tools for deciphering macrophage heterogeneity and polarization states, substantially enhancing our understanding of their complex functions in both physiological and pathological contexts. Conventional population-level analyses often mask cellular heterogeneity, whereas single-cell RNA sequencing (scRNA-seq) enables precise characterization of macrophage subpopulations and transcriptional profiles across diverse tissue microenvironments, allowing accurate identification of subsets with specific functional states or polarization tendencies. For instance, in liver cancer research, Stereo-seq technology identified a 500-micrometer-wide invasive zone at the tumor margin with nanoscale resolution; damaged hepatocytes in this region overexpress serum amyloid A, inducing M2 polarization of macrophages via JAK/STAT3 pathway activation, thereby promoting local immunosuppression and offering new insights for targeting the TME (325). Similarly, GeoMx-based spatial multi-omics analysis of postmortem pancreatic samples from COVID-19 patients revealed spatial clustering of pro-inflammatory macrophages that mediate β-cell pyroptosis through the TNFSF12/TNFRSF12A signaling axis; furthermore, a human vascularized macrophage-islet organoid model was developed to effectively simulate immune-mediated cell damage (326). Moreover, integrating single-cell and spatial omics has uncovered functional heterogeneity of macrophages under physiological conditions. In wound healing, for example, combined scRNA-seq and spatial transcriptomic analyses demonstrated that dysfunctional Spp1^+^ macrophages in aged individuals suppress type II immune responses, thereby impairing extracellular matrix-mediated repair of epidermal cells and fibroblasts (327). These technological breakthroughs not only deepen our knowledge of macrophage heterogeneity but also open new avenues for precisely modulating their polarization states.

In TME studies, multi-omics integration strategies further underscore the complexity of macrophage polarization regulatory networks. Research in colorectal cancer revealed that sphingosine-1-phosphate (S1P) promotes M2 polarization through the migration inhibitory factor (MIF) pathway in macrophages. Spatial transcriptomics confirmed that regions with high Sphingosine kinase 1 (SPHK1) expression closely correlate with the spatial distribution of M2-like macrophages, while inhibiting S1P synthesis reverses their pro-angiogenic phenotype (328). Notably, integrated immunogenomic analysis in ovarian cancer established a dynamic model of macrophage polarization. This model employed artificial neural networks to quantify the balance among M0/M1/M2 subsets and successfully predicted patient prognosis and treatment response. It demonstrated that the spatial distribution of pro-inflammatory (M0/M1) and anti-inflammatory (M2) phenotypes directly influences clinical outcomes (329). These findings not only deepen the understanding of macrophage heterogeneity and polarization mechanisms but also highlight the power of multi-omics integration in deciphering regulatory networks and predicting clinical trajectories.

### 6.2 Computational modeling

Computer modeling has emerged as a vital tool for deciphering the dynamics of macrophage polarization and its regulatory strategies, enabling quantitative predictions of complex biological processes by integrating multi-scale data. For instance, in inflammatory bowel disease research, molecular docking simulations accurately predicted the coordination behavior between tofacitinib and copper ions. This insight led to the development of dual-responsive nanoparticles, T-C@HP, which targeted the inhibition of JAK1 phosphorylation and ameliorated the colitic microenvironment by modulating macrophage polarization, thereby guiding the design of an intelligent drug delivery system capable of scavenging ROS/H_2_S (330). The integration of machine learning with high-throughput experiments has significantly accelerated biomaterial discovery. Utilizing dynamic laser interference lithography, a million-scale nanotopography array was constructed and analyzed via Gaussian process regression, identifying key topographic features that promote M2 polarization. This model further revealed that cytoskeletal remodeling and ROCK-dependent epigenetic regulation are central to macrophage phenotypic switching (331). High-throughput screening has identified approximately 300 compounds capable of selectively inducing either M1 or M2 polarization, some of which enable reprogramming between these phenotypes, offering a rich molecular repository for targeted therapies (332). Multi-channel sensor array technology allows high-throughput identification of macrophage polarization states within minutes, greatly enhancing phenotypic analysis efficiency (333). Additionally, genetically engineered bioluminescent reporter systems combined with phasor analysis of spectral imaging enable real-time dynamic monitoring of macrophage polarization in living cells and complex three-dimensional environments (334). Innovative applications of graph theory provide a mathematical framework for designing biomimetic extracellular matrices. By modeling the topology of RGD ligand networks, it was discovered that reducing the network’s shortest path enhances integrin recruitment capacity. Furthermore, tunable magnetic nanorods were used to modulate the myosin II/ROCK signaling axis, enabling dynamic conversion of macrophages toward a pro-regenerative phenotype (335). Current computational models are evolving from static descriptions to dynamic predictions. Future efforts should focus on developing multi-scale integrated models, combining single-cell omics to construct spatiotemporal maps of polarization signals, enhancing the application of deep learning in cross-scale mechanistic exploration, and employing virtual screening platforms to predict nanomaterial-immune cell interactions, ultimately advancing precision clinical translation targeting macrophage polarization.

### 6.3 Engineered macrophages

Engineered macrophage technologies have successfully overcome the functional limitations of natural cells through synthetic biology strategies, enabling precise regulation of the immune system. In cancer therapy, enhanced synthetic phagocytic receptors integrate FcRγ chain-driven CAR structures with secreted CD47 blockers, allowing macrophages to bypass tumor antigen heterogeneity. This promotes phagocytosis and cross-presentation of antigens, thereby activating CD8^+^ T cells and inducing an anti-tumor polarized phenotype (336). Biomimetic material engineering has facilitated the development of off-the-shelf artificial macrophages. These systems use M2 macrophage lysate protein-modified PLGA microspheres encapsulated with cell membranes to mimic endogenous macrophage functions. By releasing anti-inflammatory factors, they promote M2 polarization and enhance TH2 immune responses, significantly accelerating tissue repair (337). A microbial-cell chimera strategy employs a dual-engine macrophage-microbe encapsulation system (Du-EMME). RGD peptide modification improves its targeting capability. The R-GEM/VNP system loaded with attenuated Salmonella mediates sustained anti-tumor effects, while a variant carrying IFNγ-secreting bacteria effectively reverses the immunosuppressive microenvironment in multiple metastatic lung cancer models (338). Additionally, selenium-loaded nano-engineered probiotics (Se@EcN-C2/A2) achieve synergistic treatment of colitis by scavenging reactive oxygen species, promoting macrophage polarization toward the M2 phenotype, and restoring gut microbiota homeostasis (339). Genetically engineered macrophages delivering IL-12/CXFL9 reprogram TAMs toward the M1 phenotype. This increases CD8^+^ T cell infiltration and enhances the sensitivity of triple-negative breast cancer (TNBC) to anti-PD-1 therapy (340). Membrane engineering further expands the application scope. For instance, genetically engineered macrophage membrane nanodecoys anchored with the antimicrobial peptide LL-37 break the vicious cycle in the periodontitis microenvironment through cascade catalysis. They synergistically eliminate pathogens and promote osteogenic differentiation (341). Similarly, Kim-1-targeting peptide-modified M2 membrane-coated nanoparticles (KM@M@M) enable targeted delivery of NLRP3 inhibitors in kidney injury models, inhibiting pyroptosis by clearing ROS (342). Notably, a focused ultrasound-responsive optical platform (FUSION) achieves closed-loop control, using mechanoluminescence to provide real-time feedback on M1 polarization status (343). These advances are driving engineered macrophage technologies toward standardization and intelligence. Future efforts should emphasize cross-scale design, integrate genetic circuits with smart materials, develop universal cryopreservation protocols, and establish real-time monitoring systems to ultimately realize personalized immune programming.

## 7 Conclusion and perspectives

### 7.1 Summary of key mechanisms

The molecular mechanisms of macrophage polarization involve dynamic interactions among signaling networks, metabolic reprogramming, and epigenetic modifications, centered on the precise regulation of the balance between pro-inflammatory M1 and anti-inflammatory reparative M2 phenotypes. At the signaling level, M1 polarization is primarily driven by the TLR/NF-κB and IFN-γ/JAK-STAT1 axes. Pathogen-associated molecular patterns activate downstream signaling through Toll-like receptors, promoting inflammatory cytokine expression. Hypoxia-induced HIF-1α further enhances this phenotype by stimulating glycolysis and IL-1β release. In contrast, M2 polarization is initiated via the IL-4/IL-13/STAT6 axis and synergized by PPARγ/δ and KLF4, driving anti-inflammatory gene expression and FAO. Metabolic reprogramming represents another key regulatory dimension. M1 macrophages rely on glycolysis and the pentose phosphate pathway, while M2 cells prefer OXPHOS and FAO. Metabolic intermediates such as lactate, succinate, and β-hydroxybutyrate not only serve as energy and biosynthetic precursors but also directly participate in signal transduction and epigenetic regulation. For instance, lactate suppresses inflammatory genes by inducing histone lactylation like H3K18la, and succinate enhances glycolytic gene transcription through succinylation modifications, forming a metabolic-epigenetic feedback loop. Epigenetic mechanisms, including DNA methylation, histone modifications such as acetylation, methylation, and lactylation, as well as non-coding RNAs like miR-34a and lncRNAs, finely regulate the accessibility and expression of polarization-related genes. Notably, these mechanisms do not operate in isolation but form an interconnected network. For example, crosstalk exists between STAT and NF-κB pathways, PPARγ influences histone modifications by regulating metabolic enzymes, and HIF-1α forms a positive feedback loop with glycolysis to sustain the M1 state. A deeper understanding of this multi-layered regulatory network offers novel insights for targeted intervention in macrophage polarization imbalances. For instance, developing nanomedicines to modulate TLR/STAT signaling or using metabolites to influence HIF-1α activity may provide new therapeutic strategies for inflammatory diseases and cancer immunotherapy.

### 7.2 Unanswered questions

Although significant progress has been made in understanding macrophage polarization mechanisms, several key scientific questions regarding the plasticity and stability of their states remain unresolved. It is still unclear how specific phenotypes, such as the anti-inflammatory phenotype, maintain stable gene programs through epigenetic memory, such as DNA methylation or histone modifications, after the withdrawal of microenvironmental signals. Moreover, the molecular mechanisms underlying the long-term maintenance of polarized states in pathological microenvironments are not fully elucidated. For example, TAMs consistently maintain a pro-tumoral M2-like state, which may involve irreversible epigenetic reprogramming or metabolic locking mechanisms, such as exosomal lncRNA HAGLROS promoting M2 polarization though its regulatory network remains unknown. Similarly, in chronic inflammatory diseases like atherosclerosis, the persistence of pro-inflammatory M1 phenotypes and their association with glycolysis/HIF-1α positive feedback pathways require further exploration. Additionally, metabolites such as lactate bidirectionally regulate macrophage polarization stability through epigenetic modifications like H3K18 lactylation, yet their dominant effects and functional thresholds within specific microenvironments remain largely unknown.

The functional heterogeneity of macrophage populations and its impact on plasticity are often overlooked. Single-cell studies reveal that tissue-resident macrophages and monocyte-derived macrophages exhibit markedly divergent responses to identical stimuli. Whether this divergence stems from their developmental origin-driven epigenetic pre-programming, such as differential baseline chromatin accessibility of factors like KLF4 and STAT6, remains unclear. Moreover, the potential role of the deubiquitinating enzyme USP25 in stabilizing STAT6 may vary across subsets and requires further investigation. The existence of transitional or hybrid states during polarization, along with their metabolic profiles and commitment mechanisms toward terminal phenotypes, lacks support from real-time dynamic data. Finally, therapeutic strategies targeting macrophage polarization face translational bottlenecks. Although modulating TLR/STAT signaling or implementing metabolic interventions is conceptually viable, achieving tissue-specific delivery while avoiding systemic immune disruption remains challenging. For instance, targeting BRCC3 alleviates atherosclerosis but may inadvertently suppress NLRP3-mediated physiological repair processes. Metabolic reprogramming strategies could trigger compensatory activation risks. These challenges underscore the urgent need for spatiotemporally precise tools and advanced models to enable safe and effective clinical translation.

### 7.3 Translational potential

Research on macrophage polarization has revealed its central role in disease pathogenesis, providing a foundation for phenotype-targeted therapeutic strategies and demonstrating considerable potential for context-directed personalized medicine. However, current interventions—such as blocking pro-inflammatory signals or enhancing anti-inflammatory pathways—though somewhat effective, are often limited by the high heterogeneity among disease types, developmental stages, and individual patients. Genetic background, epigenetic regulation, local microenvironment, and commensal microbiota collectively shape unique macrophage response patterns, rendering one-size-fits-all treatment approaches largely ineffective. Therefore, future therapeutic paradigms must shift toward precision medicine, leveraging integrated high-throughput technologies—including single-cell sequencing, spatial transcriptomics, proteomics, and metabolomics—to systematically decipher patient-specific molecular profiles and dynamic signaling networks of macrophages in particular disease states. This enables the identification of individualized targets driving pathological polarization. Based on such refined molecular subtyping, tailored intervention strategies can be designed, such as developing small molecules modulating specific receptors or pathways, engineering nano-carriers for targeted delivery of nucleic acid therapeutics or gene editing tools, or even exploring ex vivo reprogramming and reinfusion of macrophages to precisely steer their functional polarization from disease-promoting toward protective or reparative phenotypes within specific microenvironments. Achieving this vision still requires overcoming multiple technical challenges, including designing delivery systems that accurately target specific macrophage subsets in tissues, mitigating the immunosuppressive microenvironment that impedes treatment efficacy, and establishing robust biomarker systems for real-time monitoring of therapeutic response. Despite these hurdles, with advances in artificial intelligence-powered predictive modeling and the integration of multi-disciplinary technologies, personalized macrophage-targeted therapy is poised to become a key strategy for managing inflammatory disorders, fibrotic diseases, cancer, and metabolic conditions, ultimately advancing more effective and safer precision immunotherapy.
